# Making and shaping endochondral and intramembranous bones

**DOI:** 10.1002/dvdy.278

**Published:** 2020-12-28

**Authors:** Gabriel L. Galea, Mohamed R. Zein, Steven Allen, Philippa Francis‐West

**Affiliations:** ^1^ Developmental Biology and Cancer UCL GOS Institute of Child Health London UK; ^2^ Comparative Bioveterinary Sciences Royal Veterinary College London UK; ^3^ Centre for Craniofacial and Regenerative Biology, Faculty of Dentistry, Oral and Craniofacial Sciences King's College London London UK

**Keywords:** chondrocyte, morphogenesis, osteoblast, planar cell polarity, skeletal development

## Abstract

Skeletal elements have a diverse range of shapes and sizes specialized to their various roles including protecting internal organs, locomotion, feeding, hearing, and vocalization. The precise positioning, size, and shape of skeletal elements is therefore critical for their function. During embryonic development, bone forms by endochondral or intramembranous ossification and can arise from the paraxial and lateral plate mesoderm or neural crest. This review describes inductive mechanisms to position and pattern bones within the developing embryo, compares and contrasts the intrinsic vs extrinsic mechanisms of endochondral and intramembranous skeletal development, and details known cellular processes that precisely determine skeletal shape and size. Key cellular mechanisms are employed at distinct stages of ossification, many of which occur in response to mechanical cues (eg, joint formation) or preempting future load‐bearing requirements. Rapid shape changes occur during cellular condensation and template establishment. Specialized cellular behaviors, such as chondrocyte hypertrophy in endochondral bone and secondary cartilage on intramembranous bones, also dramatically change template shape. Once ossification is complete, bone shape undergoes functional adaptation through (re)modeling. We also highlight how alterations in these cellular processes contribute to evolutionary change and how differences in the embryonic origin of bones can influence postnatal bone repair.

## Key Findings

- Compares and contrasts Endochondral and intramembranous bone development
- Reviews embryonic origins of different bones
- Describes the cellular and molecular mechanisms of positioning skeletal elements.
- Describes mechanisms of skeletal growth with a focus on the generation of skeletal shape

## 1 INTRODUCTION

The shape and size of skeletal elements determines their functional competence in locomotion, the species' mode of feeding and also enables vocalization together with the transmission of auditory stimuli. In some species, the size and shape of bones can also influence mate selection. Additionally, bones must provide protection for the brain, spinal cord, sense organs, and viscera. Therefore, it is critical that the correct shape and size of the bone is generated during embryonic development and adapted during postnatal growth. Abnormal skeletal size and shape underlies numerous pathologies. Examples include dysplasias involving excess bone which can restrict the foramina carrying the nerves as occurs in sclerosteosis or result in synostosis, the fusion of joints.^1^, ^2^ Alternatively, decreased bone growth occurs in conditions including achondroplasia, asphyxiating thoracic dystrophy and micrognathia, the abnormal shortening of the jaw.^3^, ^4^ Skeletal patterning abnormalities may be clearly manifest at birth, such as axial and appendicular skeletal defects in Robinow syndrome,^5^, ^6^ and less apparent changes in patterning may predispose an individual to secondary skeletal disorders and pathologies later in life.^6^ These more subtle changes include congenital hip dysplasia which increases the risk of hip osteoarthritis^7^, ^8^, ^9^ and small anomalies in vertebral development which can ultimately result in congenital scoliosis, the abnormal curvature of the spine.^10^ These disorders arise through genetic mutations and environmental alterations such as mechanical influences and can affect various stages of skeletal development from patterning, differentiation, growth and morphogenesis.^11^

Reflecting the various functions of protection, locomotion and even secondary sexual characteristics, the shape of bones is incredibly diverse. Different elements are typically classified into long (eg, humerus), flat (eg, sternum), short (eg, carpals, tarsals) and irregular (eg, vertebrae, scapula) bones. Among these “irregular” bones, deer antlers are an incredible example of the complex skeletal shapes that can be generated. Antlers develop branches and plates which are characteristic of the species and typically symmetrical, yet do so intrinsically without directionally inductive cues above the head.^12^ In this review, we discuss mechanisms that determine the shape of bones, comparing and contrasting development and growth between endochondral and intramembranous bones. We discuss which stages of bone development determine skeletal shape together with the cellular mechanisms and tissue mechanics involved highlighting some adaptations of these developmental mechanisms that contribute to evolutionary change. In so doing we identify core cellular behaviors which are applied sequentially or simultaneously in order to convert simple skeletal primordia into functionally relevant shapes.

### 1.1 Endochondral vs intramembranous bones: How do they differ and is this important?

**FIGURE 1:**
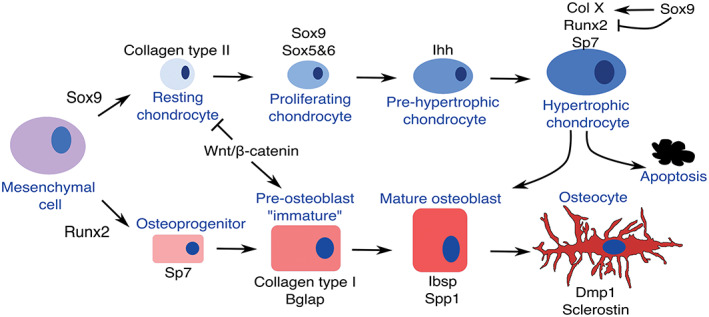
The chondrocyte and osteoblast differentiation pathways. Simplified schematic showing the key genes that are expressed during chondrogenic and osteoblastic differentiation and the relationship between the two lineages. In hypertrophic chondrocytes, the expression of SOX9 protein persists in early hypertrophic chondrocytes where SOX9 induces the expression of *Collagen type X* and inhibits RUNX2 activity. Degradation of SOX9 protein releases inhibition of RUNX2 allowing chondrocyte‐osteoblast transformation. WNT‐β‐CATENIN determines osteoblast vs chondrocyte fate in developing intramembranous bones

In bony vertebrates, bones primarily develop in two ways via endochondral or intramembranous bone differentiation. In endochondral bones, ossification occurs within the cartilaginous template and also within the surrounding fibroblastic perichondral sheath to form the bone collar. Intramembranous bones develop via direct osteoblast differentiation within the mesenchyme. Regardless of the route of development, osteoblast differentiation requires the transcription factor RUNX2 whereas chondrocyte development requires SOX9 (Figure 1).

Endochondral bones are found throughout the body. These bones arise as an initial condensation which then undergoes chondrogenic differentiation before finally ossifying. SOX9, which is expressed in the precondensing mesenchyme and chondrocytes, initiates chondrocyte commitment.^13^, ^14^ Together with the related factors, SOX5 and −6, SOX9 drives expression of early cartilaginous matrix components including *type II collagen (Coll II)* and *aggrecan* ^15^ (Figure 1). In mice loss of function of one *Sox9* allele results in smaller cartilaginous elements; similarly *SOX9* haploinsufficiency in humans results in campomelic dysplasia (bowing of the limbs) affecting all endochondral bones.^16^, ^17^, ^18^ The Sox trio (*Sox9*, *Sox5*, and *Sox6*) are highly expressed in early developing chondrocytes while terminal chondrocyte differentiation (hypertrophy) is characterized by the co‐expression of cartilaginous (*type X collagen*) and osteoblast (*Runx2*, *Sp7*, *Bone sialoprotein*) markers (Figure 1). SOX9 is also present within the early hypertrophic chondrocytes, and inhibits the function of RUNX2, which is essential for the final ossification step^19^, ^20^ (Figure 1). As SOX9 expression decreases, RUNX2 activity increases.^19^, ^20^ Thus, reduction of SOX9 expression also results in accelerated mineralization.^16^, ^19^ Depending on the skeletal element, RUNX2 alone or together with either RUNX1 or RUNX3 are also required for earlier steps of chondrogenic differentiation: for example, for the expression of *Ihh* and *Sox5/Sox6*.^21^, ^22^ For more details of molecular interactions that control endochondral differentiation, the reader is referred to Reference 14.

Intramembranous bones can be subdivided into membrane and dermal bones, which are evolutionarily part of endoskeleton and exoskeleton, respectively.^23^ In later diverged vertebrates, intramembranous bones are predominant in the head forming the cranial vault together with the majority of bones of the face. In the mammalian trunk, part of the clavicle also develops intramembraneously.^24^ In earlier diverged vertebrates, intramembranous bones are far more extensive within the trunk and include, for example, the fin rays (lepidotrichia) bones of zebrafish, and components of the exoskeleton such as the turtle plastron (ventral shell) and the small dermal bones (osteoderms, gastralia) of crocodilians.^23^, ^25^ The periosteal collar of endochondral bones is also likened to intramembranous bone. Periosteal expansion occurs through direct deposition of osteoid without a cartilage template. This physiological mode of bone formation persists throughout life and has been likened to an intramembranous‐like process, although the persistent population of periosteal progenitor cells is clearly distinct from de novo specification of osteoblasts during development.^26^

Although there are examples of dermal bones forming via metaplasia within fibrous connective tissue which lacks osteoblasts,^27^ intramembranous bones are typically formed via osteoblast differentiation. Here, initiation of osteoblast differentiation requires RUNX2 within the osteoblast progenitor which is followed by the sequential expression of the core set of osteoblast differentiation factors: *Sp7*, then *type I collagen*, *Bglap* (previously known as osteocalcin) and then *Spp1* (previously known as osteopontin)^28^ (Figure 1). Osteoblasts begin to express late markers such as *dentin matrix protein* (*Dmp)1* as they become surrounded by matrix and ultimately express the osteocye marker *sclerostin* as mature osteocytes. RUNX2 enhances the first phases of osteoblast differentiation and its function is essential within the initial progenitors and *Sp7* expressing cells.^29^ Haploinsufficiency of *RUNX2* in humans results in cleidocranial dysplasia, a syndrome that particularly affects the development of the intramembranous bones of the calvaria and clavicle which exhibit differential sensitivity to its loss.^30^, ^31^

Intramembranous bones do not involve a chondrocyte precursor as in endochondral bones where osteoblasts form within and around a cartilaginous template. In mice and chicks, the osteoblasts do express some cartilage markers such as *Sox9* and *Col II* during the initiation phase and *Col II* and *IX* mRNAs during the differentiation process.^32^, ^33^, ^34^, ^35^, ^36^ The co‐expression of cartilage markers within the intramembranous bones extends into more primitive vertebrates, frogs and zebrafish, and in these species is expanded to include the hypertrophic cartilage marker, *type X collagen (Col10a1)*.^37^, ^38^ In these earlier‐diverged vertebrates, cartilage markers are expressed at higher levels and it is hypothesized that osteoblasts evolved directly from chondrocytes.^38^ However, please note, from these studies there are mixed reports about whether the chondrocyte mRNAs are translated: it may be that there are differences between various bones and/or the osteoblast is primed ready to synthesize cartilaginous proteins when required.

The co‐expression of cartilage and bone markers indicates bipotentiality of intramembranous bone osteoblasts. Indeed, gene‐inactivation of *β‐catenin (also known as CTNNB1)*, the key intracellular mediator of the canonical WNT signaling pathway, within osteoprogenitors (and/or their descendants), or loss of WNT signaling, results in the formation of cartilage instead of bone within the calvaria dermal bones and the periosteal collar of long bones^39^, ^40^, ^41^, ^42^ (Figure 1). Similar studies have revealed that β‐CATENIN is actually required in the SP7 expressing cells and/or their descendants.^43^ This bipotential decision also requires SP7: in the absence of SP7, osteoblasts within the bone collar are replaced by chondrocytes and membrane bones within the face abnormally express chondrogenic markers.^44^ Thus, restriction of cartilage cell fate within an intramembranous bone precursor requires canonical WNT signaling. However, this restriction of cell fate does not occur at the very first step of osteoblast differentiation, that is, at the onset of RUNX2 expression, but occurs at least one step later with the expression of SP7 (Figure 1). Consistent with this the evolutionary acquisition of *Sp7*, an early RUNX2 target, is linked to the development of osteoblasts in vertebrates.^45^

Collectively, these studies indicate that there are osteochondrogenic progenitors, which co‐express *Sox9* and *Runx2* transcripts, within the developing intramembranous bones that is, they are a “chondroid” bone. Comparisons of days 12 and 17 chick calvaria have shown that chondrogenic potential of these intramembranous bones in vitro decreases in the older calvaria which is linked to decreased proliferation.^46^ Given the inhibitory effect of SOX9 on RUNX2 activity, the co‐expression of chondrogenic and osteoblastic markers may facilitate early intramembranous bone expansion combining the best of both worlds, rapid proliferation coupled with some mineralization but a delayed rate of differentiation.^19^, ^20^ Other studies have also linked chondroid bone characteristics with rapid skeletal growth in fish and avians^47^, ^48^, ^49^ while the co‐expression of chondrogenic and osteoblast markers has also been observed in regenerating intramembranous and endochondral bones.^26^, ^50^, ^51^, ^52^, ^53^, ^54^ During regeneration the source of these “hybrid” skeletal cells is the osteoblastic periosteum.^26^, ^50^, ^51^, ^53^

As will be discussed later, bipotentiality within periosteal precursors is important for development and growth of some intramembranous bones via secondary cartilages. Evolutionary it allowed a new module of skeletal development that is (a) more responsive to mechanical signals and is able to adapt to increased mechanical forces by growth—for example, during an increase in the size of jaws and (b) can develop rapidly. Intramembranous bones also differ from endochondral bones in that comparatively they contain little bone marrow, respond differentially to mechanical cues and are less susceptible to fracturing due to osteoporosis.^55^, ^56^

Thus, in answer to our initial question is the type of bone important: Yes—it is. Endochondral and intramembranous bones have different mechanisms of differentiation and growth; clinically, these differences are reflected by different susceptibility to osteoporosis and will influence surgical repair and regeneration strategies.^55^, ^56^

### 1.2 Different embryonic origins of bones: Does it matter?

**FIGURE 2:**
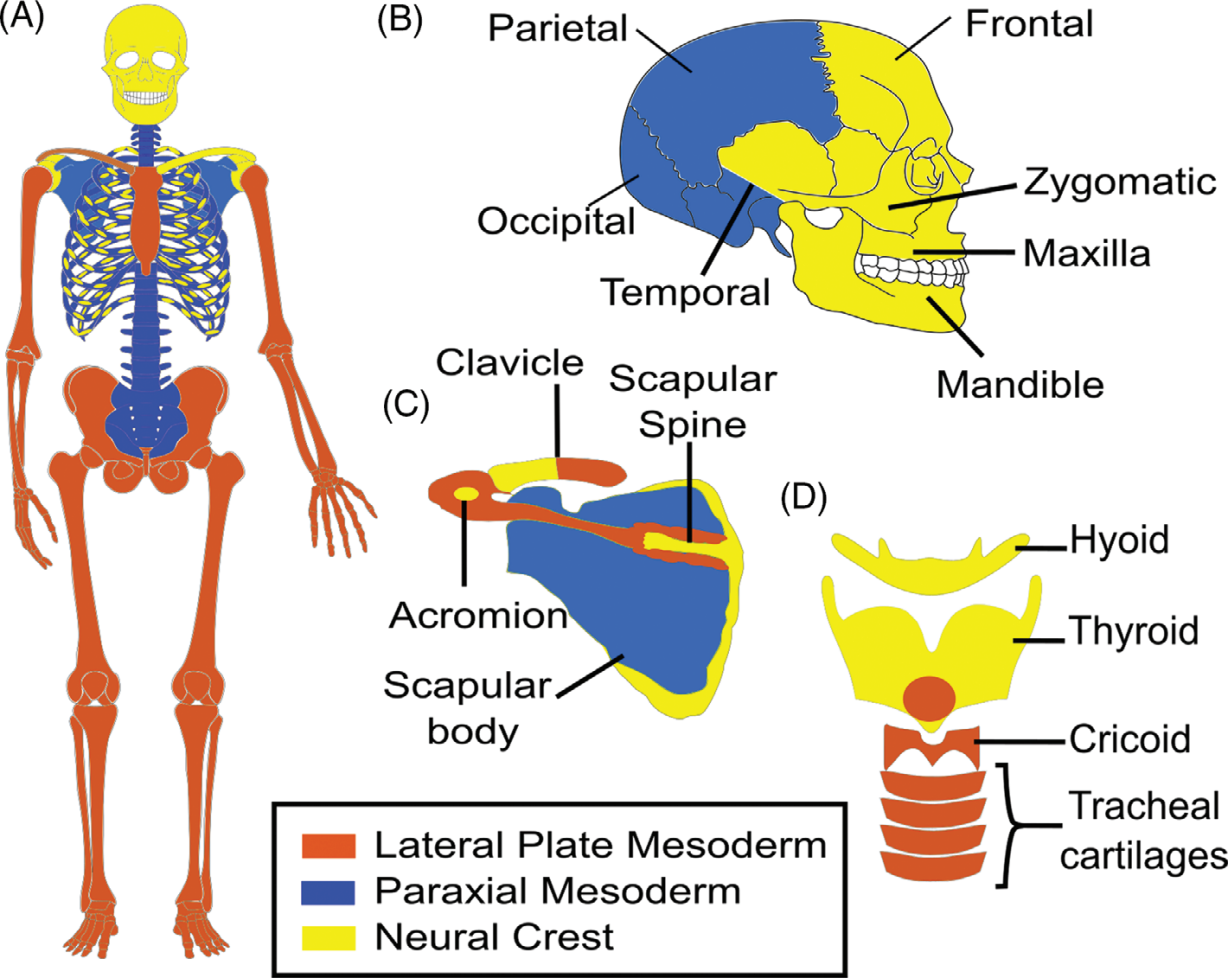
The embryonic origin of human bones. Schematic showing the proposed embryonic origin of bones from the paraxial mesoderm (blue), lateral plate mesoderm (brown), and neural crest cells (yellow) in humans based on fate mapping studies in the mouse and chick described in References 57, 58, 59, 60, 61, 62, (also see review for scapula and pelvis^63^, ^76^, ^290^). The arytenoid cartilage (not shown) also arises from the lateral plate mesoderm.^60^, ^62^ For clarity, the NCC and mesoderm contributions to the clavicle are shown separately on each side of the body in, A. B, Lateral view of head and, C,D, higher power views of the, C, scapula and clavicle and, D, laryngeal cartilages

Both the neural crest (NCC) and mesoderm lineages give rise to skeletal elements. Within the mesoderm, skeletal structures are formed from either the paraxial or lateral plate mesoderm (Figure 2). In some cases, separate condensations from these embryonic lineages integrate seamlessly to form the functional bone, such as in the temporal bone, the cranial base, the scapula, stapes, clavicle, and thyroid cartilage^57^, ^58^, ^59^, ^60^, ^61^, ^62^ (also see review for scapula and pelvis^63^) (Figure 2). Comparison of different species has indicated homologous bones can be derived from either NCC and/or mesoderm, for example, the frontal bone^64^ and heterotopic transplantation studies in the avian embryo have shown that cranial mesoderm and cranial NCC have an equivalent chondrogenic response to inductive tissues.^65^ In contrast, osteogenic capacity of NCC vs mesoderm is not equivalent: in mammals, the NCC derived frontal bone has higher osteogenic ability than the mesoderm derived parietal bone both in vitro and during regeneration in vivo.^66^, ^67^ This difference is intrinsic to the osteoblasts and is, at least in part, due to higher levels of WNT and FGF signaling, which promote differentiation,^66^, ^67^ (other intrinsic differences are reviewed here^68^). This higher osteogenic capacity may be a feature of NCC derived bones—periosteal cells from the NCC‐derived mandibular and maxillary bones are more osteogenic than periosteal cells taken from the lateral plate mesoderm derived bones.^69^, ^70^, ^71^

In all vertebrates, cranial NCC have skeletogenic potential giving rise to bone and/or cartilage.^72^ The skeletogenic potential of trunk NCC has been far less clear. Indeed, in earlier diverged vertebrates, there has been significant debate about the contribution of trunk NCC to skeletal structures but there is now strong evidence of a NCC contribution to zebrafish fin lepidotrichia and the turtle plastron.^73^, ^74^, ^75^, ^76^ In zebrafish, genetic tracing approaches have been used to follow the fate of the NCC progenitors and conclusively demonstrate a NCC contribution to fin lepidotrichia.^75^ Zebrafish trunk NCC also express molecular components of the cranial NCC mesenchymal (ie, skeletal competence) network found in later diverged vertebrates also consistent with their skeletogenic potential.^77^ In turtles, the NCC contribution to the plastron is based on DiI labeling experiments which followed the fate of a late emigrating NCC population.^73^, ^74^ Additionally, the‐expression of NCC markers within the developing skeletal elements of the plastron was determined.^73^, ^74^ A potential NCC contribution to the vertebrae and ribs was also noted.^73^ Thus, in the turtle studies there is correlation between the final destination of NCC and skeletogenesis.

In later diverged vertebrates, the classic view based on fate mapping studies in chicks and mice is that in vivo, cranial NCC, but not trunk NCC, have skeletogenic potential.^78^ Recent evidence has now, however, also shown that in mice, a small subpopulation of trunk NCC do indeed make significant contributions to skeletal structures of the axial, but not appendicular, skeleton^76^ (Figure 2). Specifically, fate mapping of the multipotent trunk NCC‐derived Schwann cell precursors aligning the nerves has shown that during a small developmental window these precursors give rise to the perichondrium and chondrocytes of the ribs and scapula^76^ (Figure 2). Again, illustrating skeletogenic potential but now following traumatic injury postnatally, another trunk NCC‐derived population (endoneurial cells within the nerves that are generated from Schwann cell precursors) can regenerate osteoblasts in response to WNT signaling from the nail bed during distal‐tip digit regeneration in mice.^79^, ^80^

In summary, both mesoderm and NCC can contribute to both endochondral and intramembranous bones. To answer our initial question: does the embryonic origin matter? Probably not for endochondral bones but a yes for intramembranous bones where NCC‐derived periosteal cells in mice have enhanced osteogenic ability.^67^, ^69^, ^71^ Of note, the identification of a NCC contribution to the ribs maybe one reason for the high regeneration capacity when compared to other endochondral bones.^52^, ^76^

### 1.3 Positioning and patterning of skeletal elements: How is this determined?

The development of some skeletal structures is induced by local signals from adjacent epithelial structures. In these skeletal elements, the initial inductive signals create the frame of the developing skeletal element. Alternatively, skeletal elements can develop within a ball or sheet of mesenchymal tissue as in the limb and calvaria, respectively. Here, how the exact positioning of the element is determined within the mesenchyme is less clear but again will depend on combinatorial signals from adjacent tissue structures. Turing reaction‐diffusion mechanisms of these signals have been proposed to determine the number and spacing of bones within the limb and calvaria.^81^, ^82^, ^83^, ^84^, ^85^ In the following sections, we will discuss examples of how local signals position and start to shape the early skeletal element, how Turing reaction‐diffusion mechanisms position developing skeletal elements within the limb and cranial vault mesenchyme and finally, how intrinsic competence within the mesenchyme influences cellular responses to inductive signals.

#### 1.3.1 *Local inductive cues and the patterning of bones*

**FIGURE 3:**
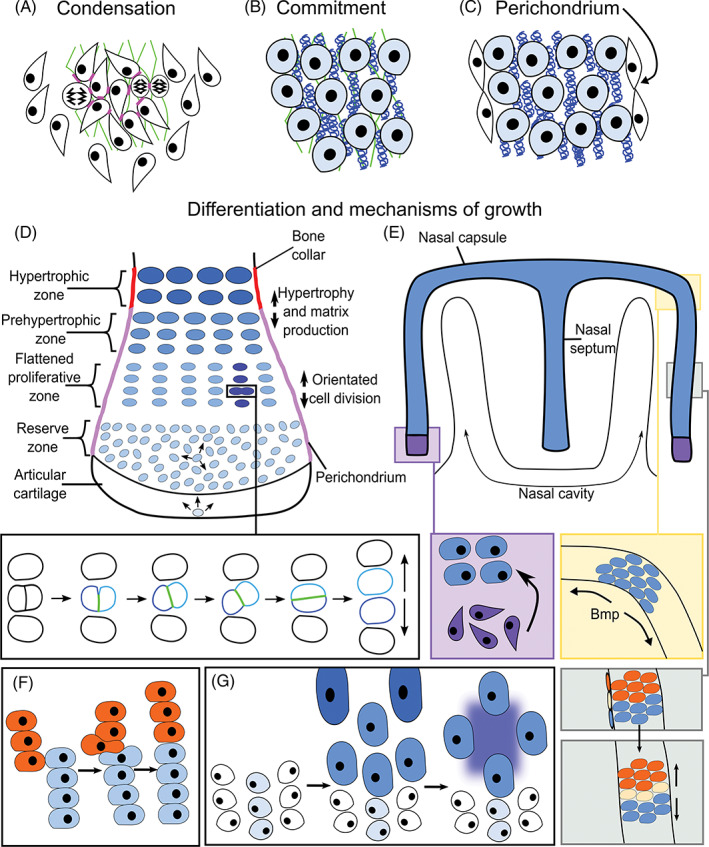
Development and elongation of endochondral bones. Phases of endochondral bone development: A, cell adhesion and ECM secretion (green lines); increased cell division may also occur, B, chondrocyte commitment and cartilaginous matrix production including collagen fibrils (blue), C, perichondrium formation, and D, establishment of the chondrocyte zones and articular cartilage. Proliferation occurs in the reserve zone, proliferating and prehypertrophic zones; orientation of cell division is indicated by arrows.^192^ In the proliferating and prehypertrophic layers, orientated divisions generate a clonal column of cells. The boxed area highlights a dividing cell shown in detail in the higher power schematic; the two daughter cells (blue) initially maintain contact through an N‐CADHERIN rich domain (green) which changes orientation until the cells finally divide.^193^, ^194^ E, Sheets and rods grow by different mechanisms.^184^ Cell behaviors within the straight and curved regions of the nasal capsule which is shaped by the adjacent epithelium. Gray box: A cell (yellow) within the perichondrium generates a column of cells across the width of the rudiment increasing its length. Orange box: Localized regions of higher BMP signaling generate a disorganized aggregate of cells from a single perichondral stem cell (shown in blue) and cause bending.^184^, ^197^ Purple box: Additional condensations can be recruited into the cartilage element.^184^ The arrows show direction of elongation. F,G, Additional mechanisms of long bone growth: (F) intercalations of short adjacent columns of proliferating cells and, G, hypertrophy, and increased matrix production (dark blue shading) increase the length of the bone. In D,F,G, the short horizontal lines indicate time; LHS, start, RHS, end of process; the orientation of long bone shown in, D

**FIGURE 4:**
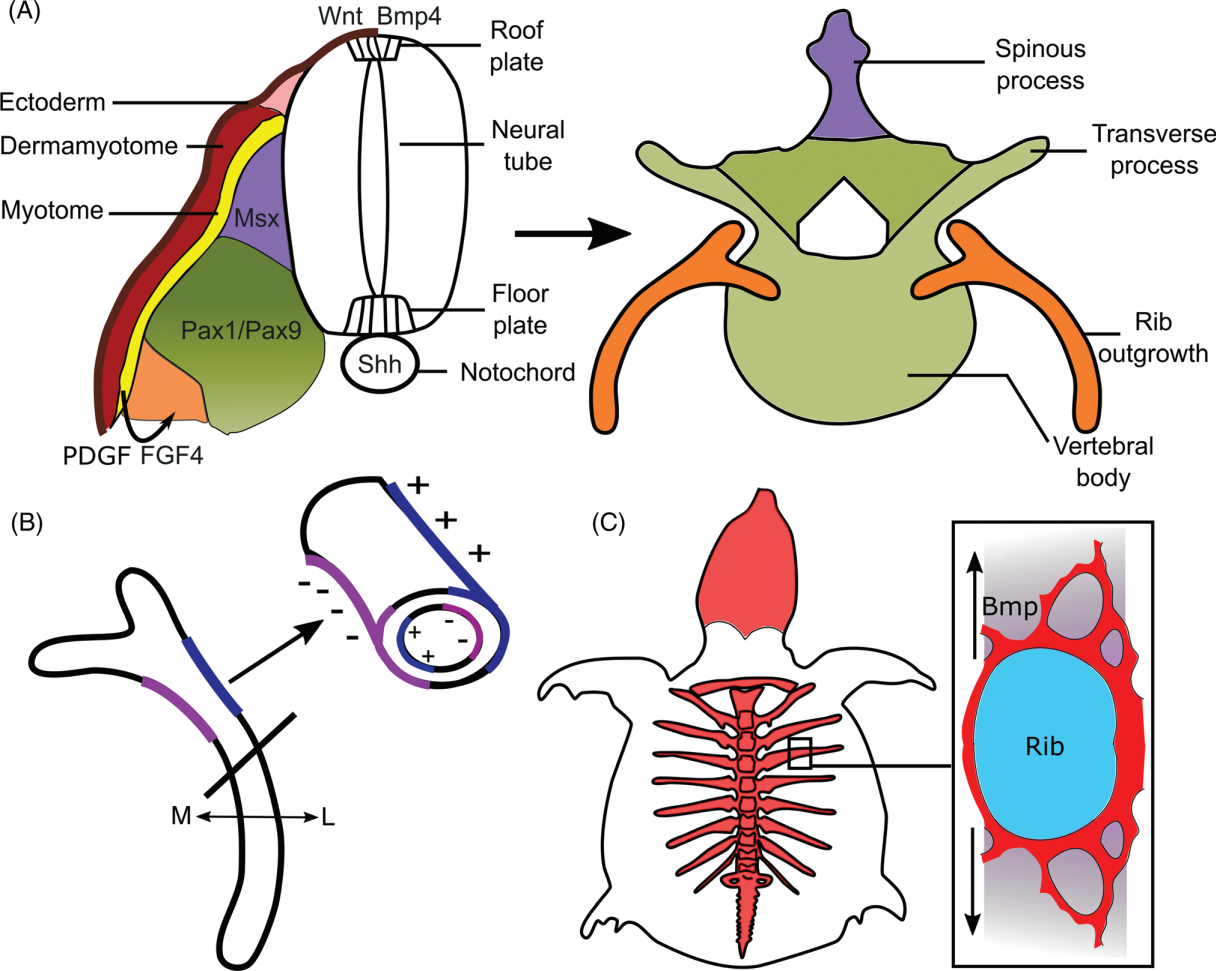
Development and an evolutionary modification of the ribs. A, The sclerotome (encompassing the green, purple, and orange domains) is specified into distinct molecular domains within the somite in response to sonic hedgehog (SHH) and BMP4/WNT signals from the notochord/floor plate and roof plate, respectively. These different domains give rise to distinct parts of the vertebrae. At thoracic levels, the early outgrowth of the rib is specified within the sclerotome in response to platelet‐derived growth factor (PDGF) and FGF signals from the adjacent myotome (yellow). B, Differential regulation of osteoblast/osteoclast activity determines growth and curvature of the ribs; osteoblast activity is regulated by BMP5.^197^ Remodeling on the periosteal and endosteal surfaces on the lateral (L) vs medial (M) sides of the ossified ribs expands the thoracic cavity. Blue shading (+), bone deposited by osteoblasts; purple shading (−), bone matrix removed by osteoclasts. C, Generation of the turtle carapace. Initially, there is a perpendicular outgrowth of bone from the rib periosteum; additional bone is then thought to be generated by metaplasia of the surrounding fibroblasts in response to BMP signaling; figure based on References 340 and 341

Many of our skeletal elements are induced and shaped by local signals from adjacent tissues which specify the complete or part of the element. Examples include the vertebrae, tracheal cartilages, the nasal and otic capsules, and the induction of the manubrium portion of the malleus by the external auditory meatus^86^, ^87^, ^88^, ^89^, ^90^ (Figures 3E and 4A). In these examples, combinatorial and/or locally restricted signals shape the skeletal element. The local induction of the manubrium which joins the body of the malleus (which forms from Meckel's cartilage) also shows how a local inductive signal can generate a more complex structure from different skeletal condensations.^87^ With a focus on sonic hedgehog (SHH) signaling, the vertebrae, cranial base and trachea will be discussed here as examples of complexity and modularity of some bones together with how regionalized signaling activity creates the specific shape of a cartilage and the timing of skeletal formation. However, please note other signaling pathways also have crucial roles and the focus on SHH is not meant to imply SHH alone is sufficient for skeletal induction.

Vertebrae are formed from somites which are initially dorso‐ventrally patterned by morphogenetic gradients of ventral SHH from the notochord/floor plate to dorsal BMP/WNT ligands from the ectoderm and roof plate^89^, ^91^, ^92^, ^93^ (Figure 4A). This patterning generates the sclerotome, the progenitor of the vertebrae, within the ventral somite and the dermomyotome within the dorsal somite. The dermomyotome then gives rise to the myotome, containing the progenitor of muscles, and dermal cells within the skin. Sclerotome cells “migrate” medially to encircle the notochord and meet their contralateral equivalents ventral to the neural tube (future spinal cord) to establish the rudiment of the vertebral body (Figure 4A). Additionally, initially looser mesenchymal cells within the sclerotome, which express the platelet‐derived growth factor (PDGF) receptor, PDGFRα “migrate” dorsally to encircle the neural tube under the control of PDGF expressed by the sclerotome^94^, ^95^ (Figure 4A). Failure of this mesenchymal “migration” precludes encircling of the neural tube and results in spina bifida occulta.^94^, ^95^ Following encircling of the neural tube, vertebrae extend vertical and transverse processes to which muscles attach. Transverse processes form in the ventral sclerotomal domain expressing the transcription factors PAX1/9 under the control of SHH, whereas the vertical spinous processes form in a MSX1/2 expression domain under the control of BMP4^96^, ^97^ (Figure 4A). This again illustrates “modularity” of skeletal development with different tissues and growth factor signals determining the development of different regions of the vertebrae (SHH, vertebral body, and transverse processes; BMP4, spinous processes) allowing the generation of more complex shapes. Other examples of skeletal elements showing molecular modularity include the scapula and pelvic girdle: like the vertebrae, the molecular modularity reflects distinct signals from adjacent tissues but additionally, it also reflects the contribution from several embryonic origins^63^ (Figure 2).

The cranial base and tracheal cartilages also require SHH for their development where, as in the somites, SHH regulates cell survival, proliferation and/or induction/maintenance of *Sox9* expression.^89^, ^98^, ^99^, ^100^, ^101^ In the absence of SHH, these skeletal elements, like the vertebrae, are hypomorphic.^98^, ^99^, ^102^ Like the vertebrae, the cranial base also develops along the midline of the embryo in association with SHH in the notochord and floor plate of the developing brain. Yet, despite the early expression of SHH, development of the cranial base is delayed relative to the trunk axial skeleton.^99^ One reason for this delay is that the cranial mesoderm is initially refractory to SHH signaling. Heterotopic grafting studies in which the trunk notochord is grafted into the head and vice versa revealed that SHH signaling is active in the cranial notochord but that the cranial paraxial mesoderm cannot initially respond to SHH signaling. Thus, the cranial notochord when transplanted into the trunk can activate the SHH pathway in the surrounding mesoderm whereas transplantation of an ectopic trunk notochord (which also expresses SHH) into the cranial mesoderm cannot.^99^ Why development of the trunk axial skeleton should be advanced relative to the head is unclear. One possibility, however, is that this delay allows the expansion of the developing brain and migration of NCC into the face before the onset of chondrogenesis, coupling development of the cranial base with that of the facial skeletal elements and the brain.^99^, ^103^

The C‐shaped tracheal cartilages develop ventrally and laterally in the mesenchyme around the tracheal epithelium.^88^ Yet, SHH is expressed throughout the dorso‐ventral axis of the tracheal epithelium indicating that the dorsal mesenchyme,^88^ which gives rise to smooth muscle cells, is either refractory to SHH signaling and/or combinatorial sets of signals are required within the ventral tracheal mesenchyme to induce/maintain chondrogenesis. Indeed, WNTS, for example, are also crucial for tracheal cartilage development.^104^ Furthermore, in explant cultures of *Shh* mutant tracheas, addition of SHH protein cannot induce skeletal development in the dorsal mesenchyme but does rescue trachea development ventrally indicating that the dorsal mesenchyme is not competent to respond to SHH by forming cartilage.^98^

#### 1.3.2 *Positioning of bones by Turing reaction‐diffusion mechanisms*

Here, we discuss the ability of mesenchyme to generate a self‐organized pattern of skeletal elements.^105^ Clues into the self‐organized patterning of chondrogenesis can be seen in limb micromass cultures which establish repeating Turing‐like expression patterns of the chondrocyte marker *Sox9*.^84^ Building on extensive in vivo evidence that WNT signaling lessens, whereas BMP signaling enlarges digits' cartilage template, a BMP/SOX9/WNT interaction model was described which reproducibly predicts the formation of five continuous digits as the limb grows.^84^ This model can explain how individual digits can emerge from a domain of uniform *Sox9*‐expressing mesenchyme. Changes in model parameters can recapitulate polydactyly phenotypes involving gains of whole digits, surplus bifurcation of individual digits, or free‐floating skeletal elements.^81^, ^82^, ^85^ Additionally, the distal *Hox* gene complex regulates the width of the digits: in the absence of almost all of the distal HOX genes, the digits are narrower and up to 14 digits can form.^85^ Finally, the size and shape of the limb autopod will determine digit number: expansion of the mesenchyme results in increased digit number.^82^ These Turing models describe how to position a condensation within the mesenchyme. Additionally, mathematical modeling combining two interdependent Turing models have been proposed to determine the position of the joints which will develop within the cartilaginous anlage.^106^

Turing models have also been applied to the cranial vault, but here they incorporate mechanical strain (defined as “percentage change in length”) caused by rapid expansion of the underlying brain in the induction of ossification centers.^83^ Osteoblastic cells are known to secrete osteogenic factors such as BMPs or prostaglandins, increase nuclear β‐CATENIN accumulation, which promotes osteoblast differentiation (Figure 1), and upregulate differentiation markers such as *Bglap* in response to substrate strain.^107^, ^108^, ^109^ In the cranial vault Turing model, strain promotes pro‐osteogenic molecule secretion and osteoblast differentiation but also alters reaction/diffusion distances to very closely resemble the pattern of calvaria formation in vivo.^83^ Altering model parameters can expand the ossification domain such that bones fuse prematurely, simulating genetic deletion of osteogenesis inhibitors which produce craniosynostosis, the premature fusion of one or more sutures.

Evidence that signals from the osteogenic front can control the patterning of cranial bones is found in zebrafish *sp7* mutants. In these mutants, cranial bone differentiation is delayed and the cranial vault is characterized by a random mosaic of bones that arise from ectopic ossification centers.^110^ Formation of multiple osteogenic centers occurs naturally in earlier diverged vertebrates and Wormian bones, small ectopic bones, are often found in humans where the fontanelles (gaps between several adjacent bones) are abnormally large such as in cleidocranial dysplasia. Ectopic bone formation is also observed in mouse mutants when cranial bone growth is delayed, for example, in Wnt1^Cre/+^ *Msx1/Msx2* ^*Fl/Fl*^ mutants.^111^ In both the Wnt1^Cre/+^ *Msx1/Msx2* ^*Fl/Fl*^ mouse and zebrafish *sp7* mutants BMP signaling, an osteogenic inducer, is increased.^110^, ^111^ Application of beads soaked in the BMP antagonist, NOGGIN, to the cranial vault of *Wnt1^Cre/+^Msx1/Msx2* ^*Fl/Fl*^ mutants prevents heterotopic ossification demonstrating that BMP signaling is necessary.^111^ A Turing model where one bone inhibits the development of another may also explain the loss of the parietal bone in *Fuzzy* mouse mutants in which there is an expansion of the frontal bone (due to the generation of excess neural crest) and the parietal bone never forms.^60^ Within the Turing model, an inhibitory signal from the larger developing frontal bone would prevent parietal bone initiation. However, as will be discussed later, while this model may explain positioning of the ossification initiation centers and evolutionary variations, mechanisms of cranial vault development in later diverged vertebrates are in fact very robust and additional mechanisms are in place to ensure correct cranial vault patterning (see 3.4, the generation of, and growth at, sutures).

#### 1.3.3 *Intrinsic regulation of skeletal competence*

Skeletal elements can have equivalent inductive signals, for example from the ectoderm or notochord during vertebrae development, but generate different shapes. Thus, precise patterning is determined by the responding tissue. This is also clearly illustrated by the differences in arm vs leg skeletal elements that arise from the lateral plate mesoderm in response to SHH, WNTs, FGFs, and retinoic acid. Chimeric epithelial‐mesenchymal studies in avians where leg mesenchyme has been recombined with wing epithelium shows that the precise shape of the skeletal element is determined intrinsically within the mesenchyme.^112^ In these chimeras, a leg develops (the origin of the mesenchyme) and not a wing (the origin of the ectoderm). Digits can even emerge from reaggregated limb mesenchyme that has been dispersed and reassembled within a limb ectodermal jacket: the identity of the digit is determined by the origin of the cells along the limb anterior‐posterior axis.^113^, ^114^ Similar studies have also demonstrated intrinsic competence of somites and the facial primordia.^115^, ^116^ Thus, NCC, paraxial and lateral plate mesoderm contain patterning information to determine the size and shape of skeletal structures in response to inductive signals.

In vitro analyses of chondrogenic differentiation in cultures of mesenchymal cells isolated from limb buds and facial primordia have confirmed that skeletogenic potential and patterning is intrinsic to the mesenchyme.^117^, ^118^, ^119^ Thus, comparisons of explants and micromass cultures between forelimb (wing) and hindlimb (leg) from mouse and chick limb buds at different developmental stages show intrinsic differences in the size of nodules and ECM production within these nodules.^118^, ^119^ These inherent differences emerge at the very first step of chondrogenesis: in chick embryos, fibronectin assembly preceding mesenchymal condensation differs between the wing and leg mesenchyme.^119^

Molecularly, these intrinsic skeletogenic differences are driven by the differential expression of transcription factors which either act cell‐autonomously within the developing skeletal element or determine the levels of paracrine signaling from adjacent cells/tissue structures. Skeletal shape determining transcription factors have been identified in the appendicular (PITX1), axial (HOX), and cranial mesenchyme (DLX5/6). The presence or absence of PITX1, which is necessary (and sufficient when misexpressed in the developing forelimb) for hindlimb development, clearly influences limb bud chondrogenesis.^118^ In PITX1‐expressing hindlimb cells, there are weaker cell/cell or cell/ECM adhesion, allowing cultures to spread over a larger area when compared to the non‐PITX1 expressing forelimb mesenchyme.^118^ Cultures of *Pitx1* null hind limbs resemble those from the forelimbs showing that PITX1 determines the different cellular behaviors.^118^ Similarly DLX5 regulates facial chondrogenesis by modulation of the expression of cell adhesion molecules, N‐CAM, and N‐CADHERIN.^120^

These intrinsic differences may not only influence embryonic patterning of skeletal elements but also postnatal growth and regeneration mechanisms. For example, developmental molecular programs are reactivated postnatally during repair of articular cartilage and calvarial bones.^121^, ^122^ Additionally, embryonic Hox gene expression patterns can be maintained within stem cell populations postnatally where they influence stem cell characteristics.^69^, ^123^ Differences in regenerative capacity of the frontal vs parietal postnatal bone are also linked to their distinct embryonic molecular signatures.^67^ Recent transcriptional analysis of stem cell responses during distraction osteogenesis of the murine mandible has also shown that one of the critical stem cell populations has the hallmarks of an embryonic NCC molecular signature.^54^ Maintenance of embryonic patterns of differential gene expression postnatally could, therefore, potentially not only contribute to intrinsic variations in the mechanisms and rates of growth between skeletal elements but also to differences in postnatal repair.

## 2 MAKING AND SHAPING ENDOCHONDRAL BONES

Shaping and growth of endochondral bones is determined by a variety of cell behaviors. First, cell polarity within the mesenchyme and cell intercalations within the condensing mesenchyme can shape the rudiment.^124^, ^125^ Following establishment of the condensation and perichondrium formation, growth and shaping is determined by orientated or localized cell proliferations, rates of cell proliferation, cell intercalations, hypertrophy and matrix production together with recruitment of stem cells from the perichondrium^126^ and other stem cell populations (for a review of skeletal stem cells, see Reference 127) (Figure 3). In most endochondral skeletal elements, formation of the perichondrium limits further recruitment of cells from the surrounding mesenchyme and growth is intrinsic to cells within the cartilage rudiment and perichondrium.

A single condensation can generate two or more skeletal elements. Individual skeletal elements can be created by generation of synovial joints from *Gdf5* expressing cells within and adjacent to the anlage.^128^ In some instances, however, chondroclast activity creates two separate bones from one cartilaginous precursor. A key example in mammals is the separation of the malleus from the transient Meckel's cartilage, a process that requires TGFβ‐activity and hematopoietic derived chondroclasts.^129^, ^130^ Generation of a separate malleus bone was a critical step in the evolution of the middle ear enabling detection of high‐frequency sounds, and concomitantly “facilitating” the evolution of the mammalian jaw joint.^131^ In contrast, fusions may also occur which alter the potential for growth: a striking example is the mammalian cranial base which arises from 14 pairs of skeletal elements.^61^ Following fusion postnatal growth is mainly due to one major growth plate, the spheno‐occipital synchondrosis.

Following ossification, the overall shape of the cartilaginous element is maintained and now growth occurs in the growth plate and by remodeling, which involves removal of bone by osteoclasts and the deposition of matrix by osteoblasts. Osteoclasts initially arise from the yolk sac and later during development from the hematopoietic system. The yolk sac derived osteoclasts are important developmentally and neonatally whereas the hematopoietic derived osteoclasts play postnatal roles.^132^, ^133^ In the following, we describe endochondral ossification in three main steps: (a) mesenchymal condensation, (b) chondrogenesis, and (c) ossification followed by subsequent (re)modeling. We highlight the cellular behaviors that influence shaping at each step and how modification of these cellular processes can contribute to evolutionary changes. In the final sections, we discuss rib development to further illustrate some of these concepts and we also discuss how the initial basic skeletal shape is modulated by the generation of joints, tuberosities, and sesamoids.

### 2.1 Getting started: The condensation stage

The first histological step of cartilage development is the formation of a mesenchymal condensation as a result of increased cell‐cell adhesion of prechondrogenic mesenchyme (Figure 3A). This adhesion involves upregulation of N‐CADHERIN^134^, ^135^ promoted by TGF‐β,^136^ although as the *N‐cadherin* null mouse has no skeletal phenotype, alternative molecules may compensate for its deletion in the mouse.^137^ Differential cadherin‐mediated adhesion leads to cell sorting and aggregation,^138^ condensing into a region of higher cell density (Figure 3A). Prechondrogenic mesenchymal cells initially secrete hyaluronan (HA), which allows them to form cell‐ECM‐cell adhesions.^139^ In the developing limb, GDF5, which is expressed in the condensation, together with WNT5a in the mesenchyme, also promote cell aggregation.^140^, ^141^ In the spontaneous *Brachypod* (*Gdf5*) mouse mutant, the limbs are considerably shorter due to the requirements of GDF5 during the condensation phase (and during later roles regulating proliferation and hypertrophy).^141^, ^142^, ^143^, ^144^, ^145^ TGF‐β signaling then switches production from HA‐rich to a predominantly fibronectin‐based matrix.^146^, ^147^ Adhesion to fibronectin fibrils is necessary for condensation and subsequent differentiation into cartilage.^148^, ^149^ Increased proliferation has also been proposed to promote condensate formation^150^, ^151^ (Figure 3A). For further details of condensation formation, please see reviews by References 150 and 151.

Condensation is followed by blood vessel regression, which produces a hypoxic environment required for chondrogenesis.^152^, ^153^, ^154^ Hypoxia helps promote chondrogenic differentiation by inducing the expression of *hypoxia inducible factor* (*Hif)‐1α* which exerts multiple effects to promote chondrocyte survival particularly at the center of the skeletal element where oxygen levels are at the lowest.^153^, ^155^, ^156^, ^157^ HIF‐1α directly increases transcription of *Sox9* to induce chondrogenic commitment and promote the differentiation program.^155^, ^157^ SOX9, in turn, induces transient *vascular endothelial growth factor* (*Vegf*) expression within the condensation which promotes angiogenesis in the surrounding mesenchyme.^152^ Given that HIF‐1α regulates *Sox9*, it is critical for endochondral development and deletion in mice results in dramatically shortened limbs with joint and sesamoid bone fusions.^155^, ^156^ The dependence on blood supply adjacent to the condensation limits the potential size of the condensation. Notably apoptosis occurs in the larger skeletal condensations within the stylopod and zeugopod and not the smaller digit condensations in the mouse *Hif‐1α* knockout demonstrating there is a limitation to the size of an early skeletal element.^156^ Thus, homologous condensations in a chick vs ostrich will start out as a similar size. A comparable size will also enable mechanisms of segmentation, that is, joint formation within the skeletal anlage to be conserved across species. Once the elements are patterned, they can generate skeletal diversity through alterations in cartilaginous growth.

#### 2.1.1 *Planar cell polarity shaping of the early condensation*

Here, we discuss how coordinated cell polarity and rearrangements can help establish the shape of some cartilage elements. These polarized events occur in the SOX9 progenitors before the establishment of the perichondrium and onset of matrix expansion and are controlled by one of two planar cell polarity (PCP) pathways: the DCHS1‐FAT4‐PCP and Wnt‐PCP pathways. The definition of PCP is the coordinated collective cell polarity or cell behaviors within a plane of tissue.^158^, ^159^, ^160^ Both of these PCP pathways coordinate the collective orientation of the chondrogenic progenitors by generation of patterned molecular asymmetries within each cell to provide a tissue polarity.

**FIGURE 5:**
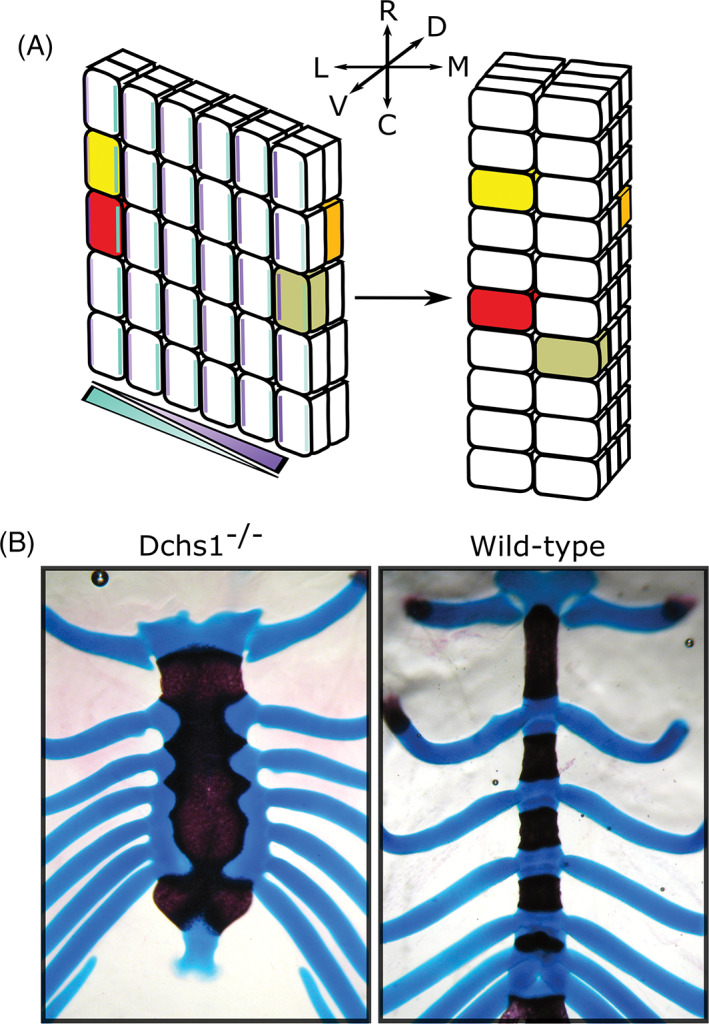
Planar cell polarity shaping of the early endochondral condensation. Schematic of FAT4‐DCHS1 regulation of sternum morphogenesis. Cells with in‐filled colors (red/yellow/orange/olive) serve as landmarks to compare time points. Initially, sternal cells are orientated along the rostral‐caudal (R‐C) axis. A gradient of DCHS1 (turquoise) and FAT4 (purple) across the medial‐lateral (M‐L) axis of the sternal mesenchyme results in higher levels of DCHS1 and FAT4 on the opposite side of each cell. This intracellular polarity is the same in each cell and results in the cells becoming collectively orientated along the M‐L axis allowing cell intercalations to narrow (across the M‐L axis), elongate (along the R‐C axis) and thicken (along the dorso‐ventral (D‐V) axis) the developing sternum. In *Dchs1* and *Fat4* mouse mutants, the cells stay orientated along the R‐C axis and cannot intercalate.^163^ B, Alizarin‐red and alcian blue staining of a *Dchs1* ^*−/−*^ and wild‐type P0 mouse sternum. Images in, B, are taken from Mao et al^164^

DCHS1‐FAT4‐PCP substantially influences the shape of the sternum. This bone forms from two condensates derived from lateral plate mesoderm on either side of the ventral midline which merge in a rostral to caudal direction.^161^ In embryos which fail to close their thoracic body wall, the sternal cartilages form but remain separated as paired bands on either side of the midline.^162^ Mediolateral narrowing, thickening along the dorso‐ventral axis and rostrocaudal elongation of the sternal rudiments requires directionally polarized intercalation movements of the prechondrocytic mesenchyme cells. This planar polarization is conveyed by graded expression of the protocadherins DCHS1 and FAT4, which act as a ligand‐receptor pair^163^, ^164^ (Figure 5A). Within each sternal cell, levels of FAT4 and DCHS1 are proposed to be highest on the lateral and medial sides, respectively^163^ (Figure 5A). This intracellular polarization of FAT4 and DCHS1 is reflected at the cellular level in the orientation of cell nuclei and filopodia of prechondrocytic condensing mesenchyme.^163^ Initially, cells are predominantly rostrocaudally oriented but under the influence of DCHS1‐FAT4 signaling, the cells reorient to have a strongly mediolateral bias (Figure 5A). This “reorientation” allows cells to move towards each other and under/on top of each other driving narrowing, thickening, and elongation of the sternum, consistent with a convergent‐extension process^163^ (Figure 5A). In the absence of FAT4 or DCHS1, cells remain preferentially oriented rostrocaudally and this convergent‐extension does not occur. Deletion of either *Fat4* or *Dchs1* in mice, therefore, produces shorter, thinner and wider sterna^163^ (Figure 5B).

Sternal shape varies considerably between species and it is possible that changes in these collective cell polarizations contribute to the different morphologies of the sternal body. For example, in emu, the shape of the sternum approximates to a square (resembling *Fat4*/*Dchs1* mouse mutants), being almost equally wide as long, in comparison to the chick where the sternum is narrower and elongated, more closely resembling sterna in wild‐type mice.^165^ Additionally, there are other differences between avians: flightless birds such as the emu have a smaller sternum (relative to thoracic cavity length), due to the assignment of fewer sternal precursors within the lateral plate mesoderm.^165^ Flightless birds also have a flatter keel, an attachment site for the pectoral muscles needed for flight.^165^

Alongside FAT4/DCHS1, PCP can also be achieved through the Wnt/*Vang*‐like (Vangl) branch of noncanonical Wnt signaling.^158^ The Wnt‐PCP pathway conveys polarity through the asymmetric localization of membrane and intracellular components such as VANGL and DISHEVELLED.^158^ WNT5a, a ligand of the Wnt‐PCP pathway is expressed at the distal edge of the elongating limb bud and directs coordinated cell behaviors. In *Wnt‐PCP* mutants, the digits are shorter, thicker, and wider; decreased length and widening of the humerus has also been reported.^6^, ^166^, ^167^, ^168^, ^169^ Digit truncation is due to decreased mesenchymal proliferation and cell survival which (a) decreases the mesenchymal progenitor pool contributing to the condensation and (b) decreases cell density which is essential for chondrogenic initiation. The widening and thickening of the digits is, however, due to loss of proximal‐to‐distal polarized cell behaviors within the mesenchyme, which result in a wider and thicker limb bud, preconfiguring the shape of the digits.^6^, ^170^ There is also the loss of coordinated cell polarity within the digit condensation in *Wnt*‐PCP mutants. Specifically, within the digits of wild‐type mice, VANGL2 localization (and phosphorylation indicative of activity) is higher on the proximal side of the cell.^171^ Every chondrocyte within the condensation therefore shows the same polarity, allowing collective cell intercalations that result in digit narrowing and elongation.

### 2.2 Chondrocyte differentiation

The condensation phase establishes an initial spatial domain where the next step, chondrogenesis, takes place. The condensate's transient fibronectin‐rich matrix is quickly replaced by a collagen (Col)‐II matrix^172^ (Figure 3B). This anlage becomes surrounded by the perichondrium which acts as a mechanical sheath and in long bones now enables elongation of the skeletal structure^173^ (Figure 3C). Histologically and molecularly, the perichondrium is not uniform around the condensation, and differential molecular signals within the perichondrium establish the chondrocytic zones and rate of chondrocyte differentiation.^173^, ^174^, ^175^ The early perichondrium also molecularly defines a straight cartilage template by secreting heparin sulfate, which counteracts BMP signaling to restrict chondrocyte differentiation.^176^, ^177^ The chondrocyte zones in the cartilage element (and later in the growth plate) consist of reservoir/resting, proliferating, flattened, prehypertrophic, and hypertrophic zones (Figure 3D).

During development, a chondrocyte transitions from a reservoir chondrocyte, to a flattened proliferative chondrocyte which then starts to enlarge through the prehypertrophic and hypertrophic stages (Figures 1 and 3D). These transitions are regulated by combinatorial groups of paracrine and autocrine growth factor signals, transcriptional networks together with systemic endocrine signals.^11^, ^14^ Examples of positive proliferative signals are PTHRP from the perichondrium, IHH from prehypertrophic chondrocytes and GDF5 from the adjacent joint interzone.^141^, ^178^, ^179^ Additionally, FGF18 signaling from the perichondrium inhibits proliferation through the FGFR3 receptor expressed in proliferating and prehypertrophic zones.^4^ Thus, the gain of function FGFR3 mutations in individuals who have achondroplasia results in fewer chondrocytes which is responsible for the significant shortening of the appendicular skeleton and cranial base.^4^ Mechanical inputs have long been known to regulate skeletal growth^180^, ^181^ and this can be illustrated by evolutionary variations. For example, lizards in hotter climates have longer limbs when compared to lizards in cooler territories and incubation of chicken embryos at decreased temperatures is sufficient to shorten skeletal elements.^182^ Higher temperatures are linked to increased muscular activity. Experimental paralysis of chick embryos has shown that muscular activity, and not the temperature, is the key determinant of the rate of skeletal growth.^183^ Furthermore, this latter study showed that a decreased mechanical stimulus from muscles increases the length of the cell cycle in some, but not all, growth plates.^183^

### 2.3 Cellular mechanisms and the shaping of cartilage

Cellular mechanisms that contribute to skeletal shape during this stage include: (a) regional proliferation; (b) the orientation of proliferation (Figure 3D); (c) the rate of proliferation; (d) cell intercalations where chondrocytes move towards each other and/or intercalate, a “convergent‐extension‐” like process (Figure 3F); (e) chondrocyte hypertrophy (Figure 3D,G); and (f) matrix production (Figure 3D,G; dark blue shading in G). Additionally, some cartilages elongate by the addition of adjacent condensations^184^ (Figure 3E and see next section). The relative contributions of each process vary between bones and species (reviewed for the limb by References 185 and 186). Examples of variations in growth mechanisms include the different rate of proliferation between the growth plates of the rat radius and tibia.^187^ Also, in juvenile zebrafish, development of some pharyngeal arch cartilages is driven mainly by proliferation with very limited contribution of hypertrophy and matrix production.^188^ Temporal differences in modes of growth may also occur. The growth rates of bat metatarsals and jerboa metacarpals do not diverge significantly from metatarsals/metacarpals in mice until the late fetal and postnatal stages, respectively, when increased hypertrophy (and increased proliferation in bats) result in their rapid elongation.^189^, ^190^

#### 2.3.1 *Orientated cell divisions*

Shape is, in part, determined by orientated cell divisions: in the long bones, growth preferentially needs to be directed along the long axis of the cartilage whereas in sheet‐like (eg, nasal capsule) or rod‐like (eg, ribs) cartilages, growth plates may not be present or sufficient to direct the appropriate longitudinal growth. In these cartilages, other mechanisms to expand the skeletal element while maintaining its thickness have evolved.^184^ Expansion and thickness are molecularly distinct processes which allows the skeletal element to grow in size while maintaining its thickness.^184^

In long bones, whereas reservoir chondrocytes orient their divisions arbitrarily, proliferative and prehypertrophic chondrocytes preferentially divide perpendicularly to the forming chondrocyte column, a feature originally identified in histological sections by Dodds^191^ (Figure 3D). The daughter chondrocytes then become integrated into the column aligning parallel to the long axis of the bone (Figure 3D). Thus, the flattened proliferating chondrocytes are stacked like coins into columns along the long axis of the bone. Additionally, cell intercalations of cells from adjacent columns contribute to elongation (Figure 3F). It was initially proposed that the reintegration following perpendicular cell division occurred via a convergent extension‐like/reintercalation process similar to the Wnt‐PCP intercalation processes in other tissues. In this case, the two daughter cells rotate around each other to align themselves within the elongating column.^192^ This consensus has recently, however, been reappraised following live‐imaging studies. In the mouse presphenoidal synchondrosis (a bidirectional growth plate within the cranial base) and in the growth plate of chick metacarpals, it was observed the daughter cells do not fully separate following mitosis.^193^, ^194^ Instead, they maintain contact via an N‐CADHERIN rich domain^193^, ^194^ (Figure 3D, and high power black box). In a process that has been described as “pivoting” the region of cell contact between the two daughter cells is “remodeled” such that it expands and in doing so becomes parallel to the long bone axis^193^, ^194^ (shown by green line in the blue dividing cell in Figure 3D). This “pivoting” repositions both daughter cells within the column before they finally undergo cytokinesis (Figure 3D and high power black box). Wnt‐PCP is essential for the initial perpendicular cell division but not for pivoting.^192^, ^194^ Pivoting may be dependent on the heterogeneity in matrix stiffness within the growth plate.^195^, ^196^ Atomic force microscopy measurements of embryonic and postnatal mouse tibial growth plates has shown that matrix in the longitudinal septa (ie, between the columns) is stiffer than the territorial matrix directly around the cells.^195^ Thus, establishment of the growth plate with its columnar structure is associated with increasing matrix production and organization of the collagen fibrillar network which may provide a mechanical framework to guide chondrocyte pivoting.^195^

Some cartilages, such as the nasal capsule, cribiform plate and ribs grow by an alternative/additional mechanism. In these cartilages, clonal fate labeling studies in mice have shown that growth occurs from a progenitor (probably within the perichondrium) which gives rise to a clone of cells that transverses the width of the cartilage^184^ (Figure 3E and high power gray box). The generation of each chondrocyte column contributes to expansion of the cartilage as the clone of cells integrates linearly across the width of the rudiment promoting elongation without changing the thickness (Figure 3E and high power gray box). Cartilage thickness is determined by the rate of differentiation, that is, when the chondrocytes cease to proliferate. Accelerating chondrocyte differentiation by deleting G‐protein stimulatory α‐subunit produces thinner cartilage sheets with shorter chondrocyte columns.^184^ Additionally, in “rods” and “sheets” expansion also can occur via recruitment of new condensations that develop adjacent to the cartilaginous element^184^ (Figure 3E, purple box). These mechanisms produce linear sheets or rods of cartilage: in regions of cartilage that bend or buckle, chondrocyte divisions are not organized and clones of cells form in aggregates or clusters^184^ (Figure 3E, orange box shows a clone of cells). These regionalized nonpolarized zones of proliferation may be regulated by levels or “hot‐spots” of BMP signaling (Figure 3E, orange box). Indeed, analysis of the *Bmp5* enhancers has identified enhancer activity specific to regions of outgrowth and buckling of the nasal turbinates.^197^ Furthermore, ectopic over activation of BMP signaling by expressing a constitutively active BMP receptor (activin receptor type I) focally disrupts column organization and instead results in chondrocyte clusters producing localized bulges within the cartilage sheet.^184^

#### 2.3.2 *Directed elongation through cell hypertrophy and ECM production*

Increases in ECM volume and cell hypertrophy (Figure 3D,G), make significant contributions to the elongation of the cartilaginous template but these changes do not occur in all skeletal elements.^188^ Hypertrophy preferentially expands the size of the cell along the long axis of the skeletal element^198^ (Figure 3G). The process of hypertrophy can be subdivided into an initial increase in proportional dry mass and fluid volume followed by rapid swelling and a final stage of proportional increase.^189^ Whether a hypertrophy progresses to the final stage depends on the species and skeletal element. Hypertrophy is most extensive in mammals and the largest hypertrophs are found in faster elongating bones, for example, in jerboa metatarsals and bat metacarpals.^185^, ^189^ Historically, analysis of histological sections and proliferation studies has indicated that proliferation, matrix generation and hypertrophy are all important for elongation. Two recent reports using live imaging to analyze cell behaviors in real time now indicate that hypertrophy and increased matrix are the primary mechanisms of elongation in long bones. Live imaging of the mouse fetal ulna growth plate was able to visualize perpendicular division of proliferative zone chondrocytes, but distal displacement of prehypertrophic chondrocytes was primarily achieved by cell hypertrophy and ECM expansion not convergent extension or pivoting movements following cell division.^199^ C‐type natriuretic peptide (CNP) is known to elongate endochondrally ossified bones of the axial and appendicular skeleton by enlarging the reserve, proliferating and hypertrophic growth plate zones.^200^ Treatment with CNP during live‐imaging primarily increased distal displacement of prehypertrophic zone chondrocytes by enhancing their increase in cell volume.^199^ This builds on a substantial prior report which dissected the contributions of quantifiable cellular behaviors to the extension of live‐imaged chick metatarsal growth plates.^201^ A combination of 3D cell tracking and in silico modeling again concluded that the increase in cell volume characteristic of hypertrophy and concomitant ECM volume expansion are sufficient to explain the elongation observed during imaging.^201^

Thus, these studies provide a critical snapshot of developmental processes and again demonstrate the importance of hypertrophy and matrix production in growth as proposed by classic histological approaches.^185^, ^186^, ^198^ A few potential limitations, however, of live imaging explanted skeletal elements must be acknowledged. Although explanted bones achieve the same increase in length as ones left in vivo over the same length of time,^201^ whether they would continue to elongate and achieve the same ultimate length is unknown. Additionally, laser‐induced phototoxicity in live‐imaging can trigger the integrated stress response,^202^ which has itself emerged as a substantial regulator of chondrocyte hypertrophy.^203^ Importantly, explanted rudiments lose polarizing and proliferative cues from surrounding tissues such as regional expression of GDF5, BMP4, WNT9a, or WNT5a.^140^, ^179^, ^204^, ^205^ ex vivo explants also fail to recapitulate the in vivo mechanical environment. Mechanical loading from muscle contraction promotes reorientation of perpendicularly divided proliferative chondrocytes and increases chondrocyte column length by cell intercalations.^206^ Finally, the relative contribution of the cellular processes that contribute to skeletal elongation varies between skeletal elements and temporally within an individual skeletal element.^185^, ^186^, ^198^ Thus, conclusions from live imaging studies must be interpreted with caution and it should be emphasized that the data apply to the one skeletal element at a particular stage of development.

### 2.4 Ossification, vascularization, and remodeling

Osteogenesis is the next step in endochondral ossification. The process of terminal chondrocyte hypertrophy and progression to bone has been studied for many years. In each long bone, ossification starts in the center of the cartilage, the diaphysis.^207^, ^208^ In mammals, secondary ossification centers form more laterally in the epiphysis at later stages.^208^ The cartilage remnant sandwiched between these ossification centers forms the growth plate which continues to elongate the bone postnatally. The zone of Ranvier flanks the growth plate between the proximal and distal osseous elements and is believed to contain osteochondral progenitors.^209^, ^210^ Ossification is initiated by chemotactic cues including VEGF release from terminally hypertrophic chondrocytes in the cartilage template's hypoxic core.^211^ VEGF has multiple effects: it recruits chondroclasts to create channels in the cartilage, stimulates angiogenesis to vascularize this template and chemotactically attracts osteoblasts, closely coupling angiogenesis and osteoblastogenesis.^211^, ^212^, ^213^ Cartilage canals have also been described in the dense secondary ossification centers and are believed to allow ingression of osteogenic cells.^214^, ^215^

The close temporal coupling between angiogenesis, cartilage resorption and osteogenesis is well established, but the nature of the vessels involved and their molecular function has only recently been described. Two predominant capillary‐like vessels initially invade the cartilage template: ones expressing high levels of CD31 and endomucin (type H) and ones with low levels of these markers (type L).^212^ Osteoblast precursors are primarily present around the much rarer type H vessels.^212^ The prevailing dogma is that osteoclast‐like chondroclasts resorb cartilage, explaining the persistence of mineralized cartilage in the bones of patients who have osteopetrosis due to osteoclast deficits.^216^ Although osteoclasts are indeed involved in vessel‐induced cartilage degradation, their primary role was recently shown to be the formation of type H vessel anastomosis, not resorption.^217^, ^218^ In fact, the type H vessels are also required for cartilage degradation through the expression of matrix metalloprotease (MMP‐9).^217^ Thus, the replacement of the cartilage template with bone involves interplay between VEGF‐expressing hypertrophic chondrocytes, blood vessels, osteoblasts, and myeloid‐lineage osteoclasts. This interplay is further complicated by the now established finding that hypertrophic chondrocytes can transdifferentiate into bone‐forming osteoblasts^219^ (Figure 1).

Following ossification, growth occurs at the growth plate and via remodeling, which can also reshape the skeletal element (discussed below for the ribs). Hormonal, transcriptional responses to growth factor signaling and epigenetic regulation of growth plate elongation largely determines ultimate bone length.^220^, ^221^ Each growth plate has a unique growth potential before chondrocyte proliferation is superseded by hypertrophy and growth plate ossification, “senescence.” Shorter bones undergo growth plate senescence earlier during development than longer bones.^186^, ^222^

### 2.5 Rib patterning and morphogenesis

Rib development is an example of how (a) mesodermal patterning is specified by differential expression of transcription factors, in this case, HOX genes; (b) localized proliferation within an early skeletal condensation can establish an initial outgrowth; and (c) how regionalized BMP signaling can sculpt the shape of a skeletal element. Additionally, as discussed earlier cell divisions that give rise to clones of cells transversely across the element contribute to rib elongation.^184^ Ribs arise from the vertebral body, within the ventral somitic mesoderm, in response to FGF and PDGFα signals from the myotome, containing the *Myf5/My6* expressing myogenic precursors of the axial muscles^223^ (Figure 4A). FGF and PDGFα promote sclerotome/chondrocyte proliferation and their expression is regulated by MYF5 and MYF6, together with SHH signals from the notochord and floor plate.^224^, ^225^ Given the key role of the myogenic determination factors, MYF5 and MYF6, rib defects often accompany abnormalities in the development of the thoracic musculature.^226^, ^227^ Following rib induction, the ribs extend into the lateral plate mesoderm under the influence of BMP signaling.^228^, ^229^

HOX genes, which are expressed in nested domains along the rostral‐caudal axis of the trunk, specify vertebrae identity, in part by the differential regulation of *Myf5* and *Myf6*.^223^ HOX6 promotes *Myf5/Myf6* expression in the hypaxial myotome which is adjacent to the rib progenitors^225^ (Figure 4A). In mice, the thoracic vs lumbar regions are characterized by the absence or presence of HOX10, respectively.^225^, ^230^, ^231^ HOX10 is inhibitory for rib formation. Loss of all *Hox10* paralogs results in the generation of ribs on the lumbar vertebrae.^230^ Conversely, gain of HOX10 function within the presomitic mesoderm inhibits rib development in the thoracic vertebrae.^232^ HOX10 antagonizes the function of HOX6.^225^, ^231^ Animals with expanded rib cages, including elephants, manatees (sea cows) and some snakes, have mutations in a critical HOX10 target site (H1 enhancer in the *Myf5* gene) which results in the loss of the HOX10 rib inhibitory activity in the lumbar vertebrae.^231^

Ribs initially are straight and then they must curve to enclose the body; this curvature must also change as the thoracic cavity expands. Curvature of the ribs in the mouse requires BMP5, whose expression is controlled by multiple conserved regulatory elements activated in spatially restricted domains within the perichondrium of skeletal structures.^197^, ^233^ These specific enhancers may not only control where *Bmp5* is expressed but the level of *Bmp5* expression potentially creating spatial domains with higher or lower BMP activity. The *Short Ear* mouse mutant, which has a loss of function mutation in *Bmp5*, is characterized, in part, by a smaller thoracic cage.^234^ Altering the spatial activity of BMP signaling also changes rib growth and curvature. Constitutive activation of BMP signaling within the lateral region of a rib under the control of one of the *Bmp5* enhancers produces mid‐body expansion of the cartilaginous rib.^197^ Conversely, expression of a dominant negative BMP receptor under the control of the same enhancer shortens and enhances curvature of the template, ultimately restricting the thoracic cavity.^197^ At birth, in mice, the ribs are ossified but continue to be reshaped (and grow) to accommodate the growing heart and lungs. Now, rib curvature is altered by remodeling, the deposition and removal of bone by osteoblasts and osteoclasts, respectively.^197^ On the lateral side of the ribs, bone is resorbed from the endosteal side while being deposited on the periosteal side (Figure 4B). Conversely, bone is deposited on the endosteal surface of the medial side of the rib and removed from the periosteal side (Figure 4B). In this way, the ribs expand laterally to enlarge the thoracic cavity.^197^

### 2.6 Appendicular synovial joints, tuberosities, and sesamoids—Mechanics is (usually) key

Ribs have some of the simplest bone shapes and functions. Many bones must provide projections for muscle attachment, such as crests or tuberosities, move relative to their partners through bending at joints, or redirect force transmission from muscle contraction as achieved by sesamoid bones. Here, we describe development of these modifications to the basic skeletal structure which are necessary for movement. Synovial joints are specified within the cartilaginous anlage whereas tuberosities and sesamoids arise from an independent cell lineage.^128^, ^235^, ^236^ All are specified independently of mechanical signals but later morphogenesis of synovial joints, tuberosities and some sesamoids requires mechanical forces.^181^

Appendicular synovial joints arise within a SOX9+ skeletal anlage by the formation of an interzone, a flattened layer of cells which will separate the two opposing cartilaginous elements.^128^ The interzone initially expresses GDF5 and BMP antagonists, such as CHORDIN, necessary to repress chondrogenesis.^141^, ^179^ The position and morphogenesis of joints is determined by signals, such as IHH and NOGGIN, from the adjacent cells within the cartilaginous rudiment.^204^, ^237^, ^238^, ^239^ Additionally, after specification, mechanical forces from adjacent muscles activate a very large number of signaling pathways that are critical for joint formation.^181^ For example, anti‐chondrogenic β‐CATENIN transcriptional activity is enriched in the developing interzone, but this enrichment is dependent on fetal muscle contraction in mice.^240^ Indeed, in the absence of *β‐catenin*, joint cavitation is compromised.^205^ In the chick, FGF2 is similarly expressed around the presumptive articular surface and FGF2 expression is upregulated by joint movement.^241^ Movement‐dependent activation of extracellular signal regulated kinase, a classical readout of FGF signaling, is required for selective mechanosensitive upregulation of HA, a water‐retaining lubrication molecule that expands the joint interzone.^242^, ^243^, ^244^ Immobilized joints also overactivate BMP signaling as indicated by increased phosphorylation of SMAD‐1,5,8 across the joint line, despite upregulation of the BMP antagonist *Noggin*.^245^ Thus, fetal movement produces a joint interzone environment characterized by high β‐CATENIN and FGF2 signaling, but low BMP signaling, favoring HA secretion and preventing chondrogenic differentiation. In the absence of movement, the joint lineage undergoes chondrogenic differentiation.^240^ The human clinical relevance of mechanics in joint formation is clearly shown by joint abnormalities including talipes in fetuses lacking muscle contraction, for example, fetal akinesia deformation sequence^246^ and clinically relevant joint incongruities in fetuses whose movement is physically restricted.^247^

Tuberosities and sesamoid bones, which arise in association with the perichondrium, may be viewed as two halves of the same coin; in fact, gene inactivation of *Gli3* in mice can transform the deltoid tuberosity of the humerus into a sesamoid.^248^ Molecularly, tuberosities and sesamoid bones initially share characteristics of both chondrocytes (SOX9) and tendons (scleraxis, SCX).^235^, ^248^, ^249^ Fate mapping studies in mice have shown that tuberosities and sesamoids arise from a distinct cell population to the initial chondrogenic lineage.^235^, ^236^ Specification of a tuberosity is determined by TGFβ signaling while outgrowth occurs in response to autocrine and paracrine BMP4 signaling from the tuberosity progenitors and the developing tendon, respectively.^250^ Subsequent tuberosity enlargement and endochondral ossification require muscle development, implicating mechanics.^248^, ^249^, ^250^ Sesamoid bones are small flat auxiliary bones (their name originates from sesame seed) typically located within tendons. These bones distribute loading and/or alter range of movement. Sesamoid bones can form from a tuberosity‐like outgrowth which detaches in a manner which is mechanically dependent, for example, the patella or mechanically independent, for example, the digit sesamoids.^235^, ^249^ Other sesamoid bones can develop independently of both the perichondrium and mechanical forces, for example, the mouse lateral fabella. ^235^ In all cases, however, like tuberosities, sesamoid bone formation still starts with SOX9+/SCX+ chondroprogenitors reflecting their association with tendon development.^235^

## 3 MAKING AND SHAPING INTRAMEMBRANOUS BONES

Intramembranous bones develop from cellular condensations which subsequently directly undergo osteoblast differentiation. As for some endochondral bones, the shapes of intramembranous bones can be relatively complex but, unlike endochondral bones, intramembranous bones can be reshaped extensively. Thus, the overall shape of an intramembranous bone is not necessarily determined during the condensation phase in contrast to the defining role of the condensation phase (once the perichondrium has formed) in the majority of endochondral bones.^151^ Please note, in this section, we focus on intramembranous bones and not periosteal bone which develops around the cartilaginous core and expands via (re)modeling.

**FIGURE 6:**
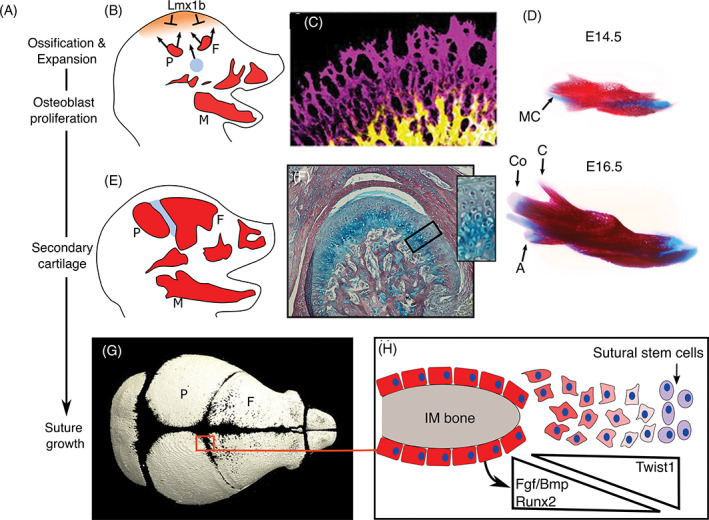
Mechanisms of intramembranous bone growth. A, Mechanisms of intramembranous growth over time. A‐D, Initially, ossifications expand by osteoblast proliferation. A,D,F, Then, in mammals and avians, secondary cartilage can develop. A,G,H, Once intramembranous bones meet, they also grow at sutures. B,E, Schematics of calvaria bone expansion and suture formation in a mouse embryo. At E14.5 (B), the frontal and parietal bones start to expand, the mesoderm that gives rise to the coronal suture (blue) starts to migrate apically. Ossification at the apex of the head is inhibited by Lmx1b (tan shading). C, Calcein (yellow) and alizarin red (purple) labeling of an E16.5 mouse frontal bone: the alizarin red staining shows the mineralization that has occurred over the previous 24 hours. D, Alcian blue and alizarin red stained developing mandibles. The mandible develops around Meckel's cartilage and by E16.5 secondary cartilages have formed on the condylar and angular processes (blue staining); the coronoid process lacks a secondary cartilage. F, Histology of a P0 mouse condylar cartilage which is a secondary and articular cartilage; note the rapid hypertrophy from periosteal/perichondral layer and disorganization of chondrocytes. G, A μCT scan of a P0 mouse skull. H, Schematic of a suture. FGF, BMP, and RUNX2 activity is higher at the osteogenic front. The transcription factor, TWIST inhibits RUNX2 activity. C,D,G, Taken from Crespo‐Enriquez et al.^296^ A, angular process; co, condylar process; C, coronoid process; F, frontal bone; IM, intramembranous bone; MC, Meckel's cartilage; M, mandible; P, parietal bone

Intramembranous bones grow via a combination of remodeling, sutural growth and via secondary cartilages, a late feature in vertebrate evolution (Figure 6A). Growth via sutures and secondary cartilage are unique to intramembranous bones. Sutures are the fibrous joints which unite adjacent intramembranous bones and contain the progenitors necessary for growth.^64^, ^251^ Secondary cartilages arise within the periosteum or in the mesenchyme adjacent to intramembranous bones to give rise to a cartilage that rapidly differentiates into bone.^36^, ^252^ Following initial ossification, osteoblasts can, therefore, arise either from the periosteum surrounding the developing bone, via secondary cartilage, or be recruited from sutural mesenchyme. Growth is achieved by cell recruitment to the osteoblast lineage together with osteoblast proliferation and extensive matrix production. As osteoblasts produce matrix they undergo dramatic morphological changes, extending elongated dendritic processes as they become embedded in osteoid (Figure 1). To date, planar polarized osteoblast behaviors such as orientated cell divisions have not been observed in vivo. However, orientated divisions in response to mechanical strain have been identified in osteoblast cells in vitro and mineralization organization is disrupted in *Wnt‐*PCP mutants.^253^ It is currently unclear if the latter reflect PCP changes in osteoblast organization or is a secondary effect.

A key take home message is that mechanical influences such as mechanical strain due to the growth of the underlying brain and eye, together with muscle activity have significant effects on the shaping and growth of many intramembranous bones. Mechanical forces influence all three mechanisms of intramembranous bone growth. The mouse coronoid process, which grows predominantly, if not solely, by modeling does not differentiate in *Myf5*/*MyoD* mutant mice that lack muscles^254^ while secondary cartilages are not induced/maintained in the absence of mechanical signals.^36^ The role of mechanical forces in also illustrated by the change in cranial vault size following alterations in the growth of the brain. The bones of a cranial vault are smaller if the brain is smaller, for example, following Zika virus infection while the cranial vault bones are larger when the brain is larger, for example, as a consequence of hydrocephaly. In the latter example, the direction of growth also appears to be altered: skulls around hydrocephalic brains are expanded and thinner. One potential mechanism could be that tension is organizing the directions of cell divisions as described in osteoblast cultures.^253^

Like endochondral bones, intramembranous bones can show molecular “modularity” which may reflect different modes of development and/or distinct molecular requirements. For example, in the *Goosecoid* mouse mutant, all parts of the mandible are reduced in size while loss of *Pax9* specifically affects the coronoid process reflecting their differential requirements in early patterning of different regions of the mesenchyme.^255^, ^256^ In the *Tgfbr2* mouse mutant, all parts of the mandible are affected but the defects in the various regions arise by different mechanisms: the hypoplastic condylar process is due to a defect in secondary cartilage while the anomaly in the mandibular body occurs because of decreased osteoblast proliferation.^257^ Modularity of intramembranous bones, and how it contributes to evolutionary change, with a particular focus on the fish opercle bone, is reviewed elsewhere.^258^

In the following, we describe intramembranous bone initiation and the three mechanisms of growth. The main focus will be on intramembranous bone development in later diverged vertebrates discussing data obtained for the calvaria and for the NCC derived facial bones. Finally, we discuss evolutionary adaptations of the rib periosteal collar in turtles and the interplay between developing intramembranous and endochondral bones. We also highlight the role of secondary cartilages in rapid bone development during antler regeneration.

### 3.1 The intramembranous bone initiation phase shares parallels with endochondral bones

As for endochondral bones, an epithelial signal is needed for initiation of many of the facial membrane bones.^259^, ^260^, ^261^ Epithelial inductive zones include the mandibular epithelium, the frontonasal ectodermal zone and possibly the endoderm, the latter two also pattern the mesenchyme to determine the arrangement of skeletal elements.^262^, ^263^, ^264^ In contrast, signals from the dura are implicated in the induction of the cranial vault bones^265^ while the position of mechanoreceptor neuromasts is linked to the ossification sites of the suborbital bones in *Astyanax* fish.^266^, ^267^ As for endochondral bones, the size of the initial cell population will influence the size of the bone: in ducks more cranial NCC are generated than in quails which will ultimately generate larger bones.^268^

Like endochondral condensations, in later diverged vertebrates, BMPs and FGFs are necessary for osteogenic differentiation acting, at least in part, via MSX1/MSX2 transcription factors which are essential for intramembranous bone development,^35^, ^111^, ^269^, ^270^, ^271^, ^272^, ^273^ (FGF signaling reviewed by Reference 274). Loss of BMP activity results in the severe hypoplasia or absence of intramembranous bone formation showing an absolute requirement^35^, ^272^, ^273^ while loss of FGF signaling delays osteogenic differentiation.^274^

As epithelial signals such as BMPs and FGFs are also required for endochondral bone development how is the cartilage vs intramembranous bone decision made? This will depend, in part, on the induction or presence of the “master” transcription factors, SOX9 and RUNX2, which are sufficient to increase cartilage and intramembranous bone formation, respectively, in vivo.^28^, ^275^, ^276^ In the developing face, SOX9 is present in the premigratory NCC whereas RUNX2 is expressed later so temporal differences in their expression can explain how two lineages can be specified by the same inductive factors.^28^

In cells co‐expressing SOX9 and RUNX2, the decision to commit to either lineage is determined by the osteochondrogenic competence of the mesenchyme which is influenced by the expression of inhibitory or activating growth factor signals, for example, canonical WNT signaling. Indeed, within the cranial vault, WNT signaling from the ectoderm is sufficient to direct osteogenic vs chondrogenic fate.^40^ Competence will also be determined by differential expression of transcriptional factors such as MSX1/MSX2 which can promote osteoblast and suppress chondrocyte differentiation.^72^, ^277^ Conversely in zebrafish, the Fox transcription factors control bipotential fate decisions to induce chondrogenesis while inhibiting the osteoblast differentiation pathway.^278^ Differential competence can be shown by FGF treatment of premigratory quail NCC which results in the formation of cartilage nodules and sheets of membrane bone reflecting the heterogeneity within the cell population.^270^ Competence can also change over developmental time which is at least partly linked to temporal differences in SOX9 and RUNX2 expression. In mice, transient ectopic FGF expression during early facial development can promote cartilage while inhibiting intramembranous bone formation.^279^, ^280^ Application of FGF at later stages within the osteogenic mesenchyme promotes osteogenic differentiation.^281^, ^282^ For further discussion about the molecular regulation of NCC skeletal differentiation, see recent review by Reference 72.

Both the intramembranous and endochondral condensations are avascular and become ossified when invaded by capillaries bringing in inorganic crystals required for mineralization; however, vascularization occurs more rapidly in intramembranous bones.^283^ Both types of condensation share cell adhesion and matrix components such as N‐CAM, tenascin (TSC), and fibronectin (FN1).^150^, ^284^, ^285^ Differentiation of both types of bone require VEGF but the mechanisms are different. Within endochondral bones, VEGF promotes angiogenesis adjacent to the condensation^152^ whereas VEGF expression within intramembranous bones has autocrine roles in osteoblast differentiation that are independent of vascularization.^286^ Compared to endochondral bone, the role of vasculogenesis is less understood in intramembranous bone development and there are very significant gaps in our understanding.^287^

### 3.2 Adding and taking away: An osteoblast‐osteoclast affair

Intramembranous bones arise as an initial ossification which expands appositionally that is, osteoblasts at the bone front proliferate to expand the developing bone. Unlike endochondral bones, where the perichondrium acts as a physical sheath restricting lateral growth, membrane bones can expand in any direction. DiI labeling and proliferation analyses of the ossification front within the calvaria have shown that early expansion is intrinsic to the osteoblasts but, later, osteoblasts are also recruited from adjacent mesenchyme.^110^, ^111^, ^288^, ^289^, ^290^ The expansion of the bone front is not necessarily uniform and differential growth will start to shape the bone: for example, the initial expansion of the frontal and parietal bones in zebrafish and mice is predominantly apically towards the top of the head^290^, ^291^ (Figure 6A‐C). Differential growth is determined by temporal and spatial variations in the rates of osteoblast proliferation and/or osteoblast density within the osteogenic fronts of the developing bone.^292^, ^293^, ^294^ The signals that drive this differential pattern of calvaria growth are unknown. In zebrafish, however, expansion of the frontal bone correlates with development of the adjacent cartilage which influences the direction of expansion.^288^ The zebrafish opercle also shows differential growth but here we have some insight into a molecular mechanism. Initially osteoblast density around the opercle condensation is uniform but, slightly later, regions of higher osteoblast number on the ventral side vs regions of more sparse osteoblast distribution dorsally can be identified.^258^, ^292^, ^295^ This variation in osteoblast number alters the shape of the opercle and is determined by the spatial pattern of *Ihh* expression which is expressed ventrally but not dorsally within the opercle.^258^, ^292^ Thus, differential localization of proliferative osteoblasts aligning the bone can play a significant role in the generation of skeletal shape. How the localized *Ihh* domain which drives this regionalized proliferation is established, however, is unknown.

Comparison of the development of quail and duck beaks, with their divergent skeletal morphology and size, has given insight into mechanisms that contribute to skeletal growth. In quail embryos, the smaller bones ossify earlier.^272^, ^275^ The earlier timing of quail bone formation is linked to (a) earlier induction of mesenchymal BMP expression which induces bone formation, (b) higher levels of RUNX2 expression, and (c) the rate of mesenchymal cell proliferation.^275^ Counterintuitively, mesenchymal cells proliferate faster in quails than in ducks.^272^ Cell cycle length and *Runx2* expression appear to be linked: increasing the cell cycle rate by misexpressing *Cyclin D1* increases RUNX2 activity.^275^ The faster proliferation rate in quails may enable cells to reach a critical density for differentiation more quickly while the higher levels of RUNX2 will promote osteocyte formation reducing the pool of proliferative osteoblasts and resulting in smaller bones—An observation made experimentally following misexpression of RUNX2 in vivo.^28^, ^275^, ^276^ RUNX2 activity is determined by its levels of expression, posttranslational modifications by signaling pathways (eg, WNT, BMP, FGF, HIPPO, FAT4‐DCHS1) together with the presence/absence of co‐factors.^296^, ^297^ RUNX2 may also be activated by mechanical stimuli and contribute to mechanoadaptive changes in osteoblast gene expression.^298^, ^299^ Changes in any of these parameters could influence skeletal morphology. Additionally, evolutionary variations in the ratio of glutamines to alanines (Q/A ratio) in a domain of RUNX2, which alters transcriptional activity, are linked to different facial morphologies in domestic dogs, bats, and some primates.^300^, ^301^, ^302^, ^303^

The activity of osteoclasts also contributes to skeletal size and morphology. A dramatic illustration of osteoclast activity in reshaping of bones is the remarkable phenomenon that occurs in the orbital bones of the Mexican blind cavefish. These orbital bones “divide” randomly and asymmetrically via the generation of channels carved out by osteoclasts.^304^ The creation of channels between the separated bones is thought to increase sensory perception by the neuromasts necessary for predation in the absence of sight.^304^ The signals that create these channels are unknown.

Osteoclasts are active from early stages of skeletal development.^305^, ^306^ Comparison of the quail and duck lower jaw has shown that the smaller developing quail bone is associated with increased osteoclast number.^305^ In the quail, there are also higher activities of MMP9 and 13, matrix degrading enzymes secreted by osteoclasts and osteocytes, respectively.^305^ Osteoblasts and osteocytes can induce osteoclast differentiation by the expression of the receptor activator of nuclear factor κb ligand (*Rankl*, also known as *Tnfsf11*).^307^, ^308^, ^309^ Thus, the bone can determine the pattern of osteoclast activity. Consistent with this, quail to duck chimeras have shown that the pattern of osteoclast activity is determined by the species of the NCC donor, that is, the quail NCC‐derived mandibular bone directs the quail spatio‐temporal pattern behavior of duck osteoclasts.^305^ Inhibition of either osteoclast activity with bisphosphonates or inhibition of MMP9 and 13 activity is sufficient to lengthen the quail jaw.^305^ Conversely activating osteoclast differentiation with RANKL shortens the quail jaw.^305^ In these studies, a differential effect on the lower vs upper jaw was noted showing that despite the equal size of the upper and lower jaw in these species, distinct mechanisms have evolved in their growth and patterning. Evolutionarily, this would be another mechanism of decoupling generation and growth of the upper vs the lower jaw in addition to the earlier differential requirements of the brain in patterning the upper jaw (the link between brain and upper face growth is reviewed by Reference 310). Differences in upper vs lower jaw length are seen in a variety of species, including avians, such as kea, and fish, such as marlin and halfbeak (for evolutionary discussions about skeletal morphology, also see Reference 311).

Thus, differential activity of osteoblasts vs osteoclasts determines the size/shape of the bone (and hollow bones, making them lighter) and can also significantly alter the shape of a bone postnatally, eg, maxilla in humans and the Mexican blind cavefish discussed above. As described earlier for the ribs, growth and/or alterations in shape/position of skeletal elements via remodeling also occurs in endochondral bones once they have ossified (Figure 4B). Despite the crucial role of osteoblasts and osteoclasts in remodeling of both types of bone, little is known about what determines their spatial activity. Osteoblast and osteoclasts are intrinsically linked through numerous coupling mechanisms which have been extensively studied in postnatal skeletal remodeling,^312^ but when in embryonic development these mechanisms first start to act is not clearly defined. These osteoblast‐osteoclast interactions may also differ: osteoclasts that play key roles developmentally and neonatally have a different embryonic origin to those that arise from the hematopoietic system and contribute extensively to postnatal remodeling.^132^, ^133^ Bone mass and architecture can also change through modeling, in which osteoblasts and osteoclasts act independently of each other as occurs during functional adaptation to loading. Loading‐engendered strain produces a continuum between preferential resorption of low‐strain surfaces and formation on high strain surfaces in the same bone.^313^, ^314^ Osteocytes play essential roles in adapting bone shape as changes in their *Sost* expression following mechanical loading direct localized bone formation.^315^ Mechanical signals known to help sculpt the facial skeleton include those caused by mastication,^254^, ^316^, ^317^, ^318^ but unknown patterning mechanisms must determine the spatial activity of osteoblasts vs osteoclasts in preparation for these load‐bearing functions.

### 3.3 Secondary cartilage takes the leading role

Secondary cartilage, a late evolutionary development, is found in mammals and birds at sites of muscle attachment and articulation^36^, ^319^ (Figure 6D, F). These cartilages can play key roles in the rapid growth of an intramembranous bone. One of the major secondary cartilages is the condylar component of the mandible (Figure 6F), which forms part of the mammalian temporomandibular joint, and contributes extensively to growth of the mandible in humans.^252^

Molecularly, secondary cartilage shares features of primary endochondral cartilages such as the expression of chondrogenic markers, SOX9, COLLAGENS TYPE II and X with the final transdifferentiation step into osteoblasts.^320^ Differences, however, include co‐expression of osteoblast markers in the chondrogenic progenitors and that proliferation occurs within the periosteal/perichondral precursors (rather than intrinsically within the chondrocytes).^36^, ^252^ Additionally, there is rapid hypertrophy of chondrocytes which results in less matrix in comparison to primary endochondral chondrocytes^36^, ^252^ (Figure 6F). Secondary cartilage also lacks the organized zones of chondrocytes in contrast to the clear separation of chondrocyte zones within primary cartilages/growth plates (compare Figures 6F and 3D). As discussed earlier, the canonical WNT signaling pathway is instructive for osteoblast vs chondrocyte fate and in the mouse postnatal condylar cartilage, WNT signaling must be repressed within the periosteal/perichondral stem cells for chondrogenesis to occur.^321^

In avians, secondary cartilages typically arise in response to mechanical force, either within the periosteum of membrane bones or as small sesamoids within the tendon.^36^, ^319^, ^322^, ^323^ In mice, secondary cartilage arises as a condensation (sesamoid bone) which secondarily unites with the membrane bone but, in contrast to avians, initiation occurs independently of mechanical forces.^254^, ^324^ In chicks the significance of mechanical forces is clearly shown by the ability of a four hourly “tug” on an isolated chick quadratojugal membrane bone cultured in vitro to induce chondrogenic differentiation: in the absence of this frequent tug chondrogenesis does not occur.^322^ The size of a muscle and its angle of muscle attachment can influence the development and/or growth of secondary cartilage in avians. This is illustrated by the presence and absence of secondary cartilage on the duck and quail surangular bone, respectively.^319^, ^323^ In the duck, the mandibular adductor muscle is larger and attaches laterally to the surangular bone whereas in the quail embryo this muscle is smaller and attaches ventrally.^319^, ^323^ Thus, quantitative and qualitative differences alter mechanical strain within the periosteum and connecting tendon progenitors. Similar differences may also explain species variations in secondary cartilage formation on the coronoid process of the mandibular bone (Figure 6D). Humans and rats, but not mice, opposums, and guinea pigs, develop a coronoid secondary cartilage.^311^ Despite the lack of a coronoid cartilage in mice (Figure 6D), *Sox9* is still initially expressed around the coronoid process indicating a potential competence to generate cartilage and explant studies have also demonstrated a transient competence when given the appropriate signals.^255^ The chondrogenic potential is there but in mice, the signals are absent or, for example, muscle activity/force is insufficient.

In contrast to the differential requirements for mechanical signals in the generation of secondary cartilages, mechanical forces are required for their maintenance in both avians and mammals. Mechanical regulation of TGFβ has been implicated in the maintenance of secondary cartilages in both avians and mice and application of TGFβ is sufficient to promote secondary cartilage in both species.^323^, ^324^ However, there are exceptions to this mechanical rule with some evolutionary quirks—the generation of cartilaginous deer antlers from the frontal dermal bone being a significant example (discussed later in section 5).

### 3.4 The generation of, and growth at, sutures

The number, together with the shape and size, of bones in the cranial vault is determined by the number of ossification centers, how fast they expand and the position of sutures which can be specified by inductive interactions or arise by “default” between any two opposing intramembranous bones. During evolution the number of cranial vault bones has been reduced considerably possibly by altering rates of differentiation and also maybe due to the presence of osteogenic inhibitory zones in later diverged vertebrates^325^ (Figure 6B). Most of our current knowledge about the positioning of bones, and the generation of sutures, has been gained from the study of the cranial vault bones in later diverged vertebrates which are separated by the coronal, sagittal, metopic, and lambdoid sutures. The focus of the following discussion will be on the coronal suture, located between the frontal and parietal bones (Figure 6G,H), which has the highest incidence of syndromic synostosis.^251^, ^326^ As a consequence, key genes required for coronal suture development and maintenance have been well characterized. However, please note the mesodermal and NCC contribution to each suture, and the precise signaling interactions that generate each suture, do vary: each suture shows differential susceptibility to gene mutations and environmental factors such as mechanical forces. For more details about suture development and mechanisms of craniosynostosis the reader is referred to the following reviews.^251^, ^326^

Although the relative mesodermal and NCC contribution to the individual bones within the cranial vault varies between laterdiverged vertebrates, there is conservation in the general patterning of the cranial vault, including the position of sutures.^64^ The order of dermal bone development is also conserved; first the frontal and then the parietal (Figure 6B). The last bone in the sequence is the occipital bone, an endochondral bone of the cranial base. The onset of ossification (relative to the rest of the body), however, varies between species, occurring earlier in mammals which is necessary to generate bones of a sufficient size to protect the larger brain.^103^ The position of the coronal suture relative to the mesoderm:NCC interface also varies: the coronal suture is located at NCC:mesoderm interface in mice, between two NCC‐derived bones in *Xenopus* and between two mesoderm bone interfaces in zebrafish and chicks.^64^ Despite these embryonic differences, molecular mechanisms of coronal suture development are at least partly conserved between fish and mammals.^64^, ^327^

As discussed earlier (section 1.3.2), a Turing reaction‐diffusion model combining strain has been implicated in the patterning and growth of the bones of the cranial vault.^83^ Consistent with this model, there is evidence that altering the rate of osteoblast differentiation changes the number of bones and/or position of sutures.^110^, ^111^, ^327^ However, in later diverged vertebrates, sutures do not necessarily arise by default at the interface between two opposing bones and inductive mechanisms can also contribute. In mice the coronal suture arises from a small group of Engrailed‐expressing mesodermal cells located just above the eye in the region called the supraorbital mesenchyme^328^ (Figure 6B, indicated by blue circle). This cell population is induced early in development by a SHH signal from the notochord and neuroectoderm and then migrates apically above the eye to finally locate between the developing frontal and parietal bones^58^, ^328^ (Figure 6E). This demonstrates instructive mechanisms contribute to the specification of the sutural mesenchyme. As the position of the initial ossification centers for the cranial vault bones and sutures appears to be conserved across later diverged vertebrates, similar inductive signals from other signaling centers in the developing brain such as from the zona limitans intrathalamica (at the telencephalon:diencephalon border) and midbrain‐hindbrain boundary (or its derivative, the cerebellum) have been proposed to also induce/maintain the coronal and lambdoid sutures, respectively.^64^, ^329^

Once the coronal suture has formed, it must be maintained. The transcription factor, TWIST, whose expression is regulated by ENGRAILED, contributes to both the establishment and maintenance of the coronal suture.^291^ TWIST regulates ephrin signaling (via regulation of *EPHA4* expression), which is essential to maintain this sutural boundary.^291^, ^330^ In mice, fate mapping of *EPHA4* mutant cells has shown that the mutant osteoprogenitor cells within the frontal and parietal bones move ectopically into the suture.^291^, ^326^ In mice and humans, the frontal bone and parietal bones are derived from neural crest and mesoderm, respectively.^58^ The different origins of the frontal and parietal bone provide an additional mechanism of coronal suture development and maintenance of patency making development of this suture more robust. Here, ephrin B1 (EFNB1) which is specifically expressed in the NCC, and not the mesoderm, helps keep the NCC and mesoderm populations apart.^331^ *EFNB1* is a X‐linked gene and mutation in females results in craniofrontonasal syndrome which is characterized, in part, by synostosis of the coronal suture.^331^ X‐inactivation in females will result in a mosaic of EFNB1‐expressing and EFNB1‐mutant cells which have different cell adhesion properties. This results in cell sorting/segregation of EFNB1‐expressing and nonexpressing cells which is proposed to result in ectopic cell movements across the suture.^331^, ^332^ In contrast, hemizygous males, where all the NCC will be equivalent and do not missegregate/mislocalize, are unaffected. Thus, this synostosis is due to a failure to maintain the NCC:mesoderm interface.

Finally, additional mechanisms are also in place to reduce the number of cranial vault bones in mammals. The reduction in the number of ossification sites is achieved by an osteogenic inhibitory zone where the mesenchyme is intrinsically refractory to osteogenic signals. Specifically, the cranial mesenchyme at the apex of the head expresses the transcription factor, LMX1b, which inhibits osteogenesis via an unknown molecular mechanism^333^ (Figure 6B). In *Lmx1b* mouse mutants, heterotopic bone formation occurs along the midline of the head.^333^

Once sutures have formed, they provide the stem cell progenitors necessary for growth—osteoblast cells are now recruited from cells within the suture to expand the bone.^289^ Three overlapping multipotent stem cell populations which contribute to growth of the bone have been identified within the *Gli1*‐, *Prx1*, and *Axin‐2*‐expressing cells found within the suture^29^, ^334^, ^335^ (Figure 6H). As in early developing intramembranous bones, osteogenic differentiation within the suture is controlled by a balance of inductive vs inhibitory signals. Suture patency, which varies between sutures and species, is maintained by signals from the dura.^265^, ^336^, ^337^ Acceleration of osteogenic differentiation—for example, due to constitutive activation of FGFR1, 2 or 3, higher levels of RUNX2 activity (eg, through gene duplication) or loss of RUNX2 inhibitors (eg, TWIST1) or depletion of the stem cell populations—will all result in craniosynostosis^326^, ^334^ (Figure 6H). Mutations in FGFR1, 2, 3, and TWIST1 are among the leading causes of craniosynostosis.^326^

## 4 TALES OF THE TURTLE

Superficially, the domed carapace (dorsal shell) of turtles and tortoises is not dissimilar in shape to the domed cranium, yet these convergent shapes are achieved through entirely different mechanisms. Periosteal rib rostrocaudal expansion underlies the evolutionary origin of the carapace. In hard‐shelled turtles both the carapace and plastron (ventral shell) are made up of outer horn‐like scutes and inner bone.^338^ The process of carapace patterning starts with a circumferential carapacial ridge of thickened ectoderm between the developing limbs, which fulfills an organizer role similar to the limb bud AER.^339^ The carapacial ridge limits the lateral growth of the ribs and prevents them projecting ventrally.^339^, ^340^ After the ribs have grown laterally and formed a rod‐like cartilaginous core, a periosteal bone collar forms around this core. Bony trabeculae then extend rostrally and caudally from this periosteum through intramembranous ossification^341^ (Figure 4C). Initially this bony outgrowth is from the periosteum. Later dermal bone is induced and recruited from the surrounding fibroblasts (probably by metaplasia) in response to BMP signaling from the rib hypertrophic chondrocytes.^340^, ^342^ In hard‐shelled turtles, intramembranous bone formation is far more extensive than in their soft‐shelled relatives. Thus, the carapace is derived from both endochondral and intramembranous development; the latter appears to be formed by two processes—initially from osteoblasts and later from fibroblasts by metaplasia.

The mechanism of carapace development is distinct from that by which the plastron forms ventrally where mesenchymal condensates produce bony contributions to the plastron directly through intramembranous ossification. As in later‐diverged vertebrates, the intramembranous condensation initially expresses *Sox9* and *Runx2*.^343^ In the turtle plastron, the sternal cartilages do not form, and it has been proposed that the plastron membranous bones, which develop when the sternal cartilage would be expected to form, are inhibitory for sternal development.^343^ Indeed, there is evidence in chick calvaria bones, that paracrine signals from intramembranous bones can be inhibitory for chondrogenesis and a similar mechanism may occur in the turtle. Specifically, fractionation experiments in which calvarial cells of E14 chicks have been separated according to size and density identified cell fractions with osteogenic potential that could inhibit chondrogenesis of limb mesenchymal cells.^344^ Conditioned media derived from these osteoblasts was also inhibitory indicating that paracrine signals from the osteoblasts have the capacity to inhibit cartilage formation.^344^

While there is some evidence that similar chondrogenic‐repressing mechanisms may occur in the calvaria, intramembranous bones clearly do not always inhibit the development of adjacent endochondral bones. As discussed earlier some bones, such as the clavicle and temporal bone, develop from both endochondral and intramembranous elements.^24^, ^58^, ^61^, ^290^ Indeed, the endochondral portion of the clavicle does not develop in the absence of the intramembranous bone component—this interdependence may reflect the requirement for paracrine signals from the intramembranous bone and/or the requirement for sufficient cell number/density to drive skeletal development.^24^ Also, in contrast to turtles, other reptiles have both membranous gastralia and a cartilaginous sternum. In these reptiles, the gastralia develop after the sternum and it is hypothesized by Rice et al that this temporal difference may allow the development of both intramembranous and endochondral bones.^343^ For example, after chondrogenic initiation and perichondrium formation, endochondral bones may become refractory to inhibitory signals from the developing intramembranous bones, allowing either the development of separate intramembranous and endochondral elements or the composite development of bones.

## 5 DEER ANTLERS: THE POWERS OF REGENERATION

Secondary cartilages are linked to rapid growth of intramembranous bones. This is clearly demonstrated by the exceptional growth rate of deer antlers which are cartilaginous structures that develop from periosteal cells of an intramembranous bone. Remarkedly and distinct from other secondary cartilages, however, skeletal shape is determined intrinsically within the periosteal cells. Antlers are secondary sexual characteristics in deer that are only present in males, apart from caribou, that are used for fighting and asserting dominance when competing for mates during the breeding season. Antlers are the fastest growing skeletal structure and the only mammalian organ that is able to fully regenerate. Antler shape is distinct from species to species, for example, antlers can be purely spiked tines as in red and roe deer or can be primarily palmate as in fallow deer and elk. Furthermore, in many species of deer, the antlers develop complex branched structures, which are reproduced year on year, demonstrating inherent patterning mechanisms exist within these tissues to generate symmetrical antlers.

In all species, the antlers develop on the frontal bone of the cranial skeleton from a specialized structure called the pedicle. Although antlers are not produced until the first year of life, the pedicle begins to develop in utero as a thickening of the periosteum on the crests of the frontal bone.^345^ The ability of this tissue to form definitive pedicles and antlers has been investigated using transplantation studies where periosteum from this region has been surgically moved to the leg^346^, ^347^, ^348^ or xenotransplanted into nude mice.^348^ These studies clearly demonstrated that these two regions of frontal bone periosteum, but no other, were able to produce a pedicle and generate (and regenerate) an antler at ectopic locations. Furthermore, it has been demonstrated that this tissue is prepatterned as rotation of the antlerogenic periosteum by 180° results in antlers that are reversed^349^ and deletion/transplantation of specific regions of the field results in loss/production of different structures.^350^ This demonstrates that at a point during development specific regions of periosteum are induced to be “antlerogenic” and are then prepatterned.

This regeneration of antlers is a mix of intramembranous and endochondral ossifications. The initial phase of regeneration involves the repair of the pedicle following loss of the previous year's antler and is via intramembranous ossification.^351^ Once this pedicle repair has been completed, the new antler proper is produced by endochondral ossification from the periosteal cells of the pedicle, a highly proliferative layer with the potential to differentiate into chondrocytes or osteoblasts depending upon external signals. Given that antlers are produced post development of the other skeletal structures and growth is predominantly endochondral arising from an intramembranous bone they are a form of secondary cartilage. However, antler development does not require a mechanical input for proper differentiation/maintenance.

The differentiation process as a whole is largely comparable to that seen in embryonic long bones but growth is appositional and a growth plate does not form.^12^, ^352^, ^353^ Molecular profiling has shown that the developing antlers (a) express NCC stem cell markers that are also seen in the stem cell populations during distraction osteogenesis,^54^ (b) resemble osteosarcoma having high levels of the expression of oncogenes which drives the rapid proliferation but in this case, is also complemented by high levels of tumor suppressors preventing oncogenesis.^354^ Another fundamental difference between antler vs long bones is the vascularization: developing cartilage in the long bone is avascular compared with that in the antler which is highly vascularized. Although it has not been investigated directly it is likely that the antler chondrocytes are not hypoxic which could be an adaptation to allow for the speed of growth required in antlers, which can be as much as several centimeters per day, and the associated requirement for oxygen and nutrients. Additionally, antler development and regeneration requires innervation, a feature of regenerating organs in earlier‐diverged vertebrates.^355^, ^356^ These incredible structures serve as an experiment of nature demonstrating how bone can be shaped, and regenerated, independently of adjacent structures.

## 6 CONCLUSIONS, FUTURE DIRECTIONS, AND CHALLENGES

The variety of structures adapted to unique functions in different vertebrates is testimony to the versatility and modularity of skeletal shape determination. Here, we have brought together studies describing mechanisms by which skeletal elements are assigned a position such as through extrinsic induction by preexisting tissues, directionality through morphogen gradients and planar polarizing cell signaling, and size at least in part through the relative cellular differentiation and ECM‐secretion rates. Recurring themes are apparent, such as the role of cell polarity in instructing the direction of growth of the limb long bones, the sternum and some endochondral craniofacial elements. It is also clear that many of these modules of cellular behavior are not unique to mesodermal vs NCC‐derived bones but that these different tissues' origins may determine rates of growth and repair. Cartilage is evolutionarily older than bone^357^ yet these cell lineages share common progenitors and are now known to be plastic. Chondrocytes can transdifferentiate into osteoblasts while osteoblasts express chondrogenic markers and have chondrogenic potential. There are, however, mixed reports as to whether the cartilaginous mRNAs are translated in intramembranous bones: what are the exact advantages of these “hybrid” chondrocyte/osteoblast cells and if there are variations in translation of “chondrocyte” mRNAs how is this regulated? Postnatally, osteoblast‐osteoclast relationships are well understood but developmentally, there are still very large gaps in our knowledge. The recent identification of distinct embryonic population of osteoclast progenitors required within the fetus and neonatally may identify novel mechanisms of bone remodeling and osteoblast‐osteoclast interactions.^132^, ^133^

Many unanswered questions persist in relation to the evolution of skeletal shape. Answering these questions may lead to novel regenerative therapies, as well as improved physical or pharmacological therapies for the large number of skeletal dysplasias. They will require the development of new research methodologies. Classical embryology experiments in avian, fish and mouse embryos were critical in describing the sequence of cellular events which initiate bone formation. Mouse transgenic models have then dissected the temporal‐spatial roles of the signaling mechanisms now widely accepted to be involved, although these models sometimes need to be interpreted with caution. Global transgenics often have multiple physiological abnormalities and it is not surprising that smaller mice have smaller bones. The use of conditional gene deletion through tamoxifen administration is limited because tamoxifen is fetotoxic^358^ and promotes early growth plate closure.^359^ Osteoblasts, osteocytes, perichondral cells, chondrocytes, osteoclasts, and blood vessels are all involved to various extents in different skeletal regions but the relative contributions of different cell populations to shape determination still remains poorly understood. Global and inducible promoter drivers can lineage trace these distinct cell populations to address these unanswered questions^360^, ^361^ but again should be used with caution, as Cre recombinase can affect ossification^362^ The contribution of each cell type is also being better understood partly thanks to improving imaging technologies, including live‐imaging of developing skeletal elements and tissue clearing allowing 3D imaging through bone.^363^, ^364^

Finally, can we integrate the molecular data that has been harnessed over the last few decades with precise changes in cell behaviors that sculpt the shape and size of developing bones. Humans have approximately 300 bones at birth, which will share core modules of skeletogenesis but each will also have a unique molecular characteristic. How do their unique features influence postnatal growth and repair? Does the normal range of variability in shape‐determination during development influence the risk of degenerative conditions in later life? Integration of intrinsic and extrinsic cellular and molecular mechanisms by which the shape of a functionally competent skeleton is specified, patterned, built, adapted, maintained, and in some cases regenerated, will continue to be a topic of fruitful research.

## CONFLICT OF INTEREST

The authors declare no conflicts of interest.

## AUTHOR CONTRIBUTIONS

**Gabriel L. Galea**, **Steven Allen**, and **Philippa Francis‐West**: Wrote the review and designed the figures with contributions from **Mohamed R. Zein**. **Mohamed R. Zein** together with **Gabriel L. Galea** prepared the figures.
